# Impact of plants on the diversity and activity of methylotrophs in soil

**DOI:** 10.1186/s40168-020-00801-4

**Published:** 2020-03-10

**Authors:** Michael C. Macey, Jennifer Pratscher, Andrew T. Crombie, J. Colin Murrell

**Affiliations:** 1 School of Environmental Sciences, University of East Anglia, Norwich Research Park, Norwich, NR4 7TJ UK; 2 AstrobiologyOU, Faculty of Science, Technology, Engineering and Mathematics, The Open University, Milton Keynes, Buckinghamshire MK7 6AA UK; 3 The Lyell Centre, School of Energy, Geoscience, Infrastructure and Society, Heriot-Watt University, Research Avenue South, Edinburgh, EH14 4AP UK; 4 School of Biological Sciences, University of East Anglia, Norwich Research Park, Norwich, NR4 7TJ UK

**Keywords:** Methanol, Rhizosphere, Stable isotope probing, Methylotroph, Methanol dehydrogenase

## Abstract

**Background:**

Methanol is the second most abundant volatile organic compound in the atmosphere, with the majority produced as a metabolic by-product during plant growth. There is a large disparity between the estimated amount of methanol produced by plants and the amount which escapes to the atmosphere. This may be due to utilisation of methanol by plant-associated methanol-consuming bacteria (methylotrophs). The use of molecular probes has previously been effective in characterising the diversity of methylotrophs within the environment. Here, we developed and applied molecular probes in combination with stable isotope probing to identify the diversity, abundance and activity of methylotrophs in bulk and in plant-associated soils.

**Results:**

Application of probes for methanol dehydrogenase genes (*mxaF*, *xoxF*, *mdh2*) in bulk and plant-associated soils revealed high levels of diversity of methylotrophic bacteria within the bulk soil, including *Hyphomicrobium*, *Methylobacterium* and members of the *Comamonadaceae*. The community of methylotrophic bacteria captured by this sequencing approach changed following plant growth. This shift in methylotrophic diversity was corroborated by identification of the active methylotrophs present in the soils by DNA stable isotope probing using ^13^C-labelled methanol. Sequencing of the 16S rRNA genes and construction of metagenomes from the ^13^C-labelled DNA revealed members of the *Methylophilaceae* as highly abundant and active in all soils examined. There was greater diversity of active members of the *Methylophilaceae* and *Comamonadaceae* and of the genus *Methylobacterium* in plant-associated soils compared to the bulk soil. Incubating growing pea plants in a ^13^CO_2_ atmosphere revealed that several genera of methylotrophs, as well as heterotrophic genera within the *Actinomycetales*, assimilated plant exudates in the pea rhizosphere.

**Conclusion:**

In this study, we show that plant growth has a major impact on both the diversity and the activity of methanol-utilising methylotrophs in the soil environment, and thus, the study contributes significantly to efforts to balance the terrestrial methanol and carbon cycle.

Video abstract

## Introduction

The large amount of carbon released to the soil via the roots of growing plants (1–20% of total photosynthate [1]) has a profound impact on the microbial communities in soil [2]. Root exudates include organic acids, sugars, alcohols, mucilage, sloughed off cells and methanol [3, 4]. Growing and decaying plants account for the majority of methanol produced globally (149 Tg year^−1^), released following demethylation of pectin in the walls of restructuring plant cells [5]. In the atmosphere, methanol is the second most abundant organic gas (0.1–10 ppb) after methane (1800 ppb) [6], but there is a large disparity between the estimated amount of methanol produced and the amount entering the atmosphere. This suggests that plant-associated methylotrophic microorganisms may be responsible for oxidation of a substantial proportion of the methanol produced by plants before it can escape to the atmosphere [7].

Methanol-oxidising methylotrophs can utilise methanol as a sole source of carbon and energy and are widespread in the terrestrial environment [7]. Methylotrophs detected in soil environments in previous studies typically belong to the Proteobacteria, although others, including *Verrucomicrobia*, *Firmicutes*, *Flavobacterium* and *Actinobacteria*, have also been detected [8–11]. Previous studies have indicated that methylotrophic bacteria are enriched in the rhizosphere of certain plant species, for example, *Methylobacteraceae* and *Hyphomicrobiaceae* in the rhizosphere of *Arabidopsis thaliana* [12], *Methylophilaceae* and *Comamonadaceae* in the pea rhizosphere and *Methylophilaceae* and *Methylocaldum* in the wheat rhizosphere [13]. Methanol dehydrogenase genes have been detected in the rhizosphere of rice, grasses, soybeans, cereals and pea plants [14–16], methanol dehydrogenase enzymes have been detected in the rhizosphere soils of oat, wheat and *A*. *thaliana* [17], and soils in association with *A*. *thaliana* had higher rates of methanol dissimilation than non-plant-associated soils [18]. However, the reasons for changes in the abundance of methylotrophs in the soil in response to plant growth are hard to identify, since many of these methylotrophs can also use multi-carbon compounds, which could be supplied either directly from the plant or from the exudate-induced accelerated breakdown of recalcitrant soil organic matter (SOM) [19].

The oxidation of methanol to formaldehyde requires the enzyme methanol dehydrogenase. There are several methanol dehydrogenases that have been characterised in different classes of methylotrophic organisms, and the most well characterised is the canonical MxaFI [20]. This enzyme is heterotetrameric in structure, with *mxaF* and *mxaI* encoding the large and small subunits respectively [21]. The large subunit contains a pyrroloquinoline quinone (PQQ) cofactor and a calcium ion [20, 21]. The function and expression of this methanol dehydrogenase in *Methylobacterium extorquens* AM1 requires 25 genes [22]. More recently, a lanthanide-dependent rather than calcium-dependent methanol dehydrogenase, XoxF, has been discovered [23]. Comparison of *xoxF* genes in a range of methylotrophs showed that there are five distinct phylogenetic clades of *xoxF*, thus representing a considerable diversity of *xoxF*-dependent methanol dehydrogenases in bacteria [24]. The XoxF methanol dehydrogenase has a specific cytochrome C_L_ and periplasmic solute binding protein associated with it, encoded by *xoxG* and *xoxJ* respectively [23]. Mdh2 is a recently discovered divergent PQQ-methanol dehydrogenase, thus far identified in two genera of the *Burkholderiales* [25]. Sequence-based analysis of Mdh2 showed that it was closely related to type I alcohol dehydrogenases rather than a highly divergent *mxaF* or *xoxF* [25].

Most cultivation-independent studies investigating the diversity of methylotrophs in the terrestrial environment have used universal 16S rRNA gene sequencing, rather than analysis of methanol dehydrogenase genes. However, there are significant issues with the use of the 16S rRNA gene to infer function, especially with methylotrophs. For example, only a few members of the genera *Bacillus* and *Flavobacterium* are methylotrophs [11, 26]. DNA-based diversity studies of methylotrophs therefore require use of a functional marker gene. Previous studies in the soil environment, using PCR primers targeting the large subunit encoding gene of the canonical methanol dehydrogenase, *mxaF* [8], have revealed a relatively low diversity [18, 26, 27], highlighting the necessity to incorporate the recently discovered novel MDH genes and enzymes, notably *xoxF* and *mdh2*, into analyses of methylotrophs in soil. PCR primers targeting different clades of *xoxF* are now available [28] but have not been extensively used in soil environments. To our knowledge, no PCR primers have been used to target the *mdh2* gene in soils. Another approach for characterising a specific metabolic guild within an environment is stable isotope probing (SIP), which tracks the incorporation of specific isotope-labelled substrates into target microbes [10]. A SIP-based approach to identify microbes actively utilising plant exudates in the rhizosphere involves incubating growing plants with ^13^CO_2_ and then identifying ^13^C-labelled carbon in DNA extracted from the rhizosphere [29].

In this study, we aimed to examine the diversity of methanol utilisers, including those that may utilise the recently discovered MDHs XoxF and Mdh2, in the rhizosphere of two common crop plants, pea (*Pisum sativum* var. Avolar) and wheat (*Triticum aestivum* var. Paragon), and to verify that these methylotrophs used plant root exudates. Firstly, DNA-SIP with ^13^C-labelled methanol was used to identify methylotrophs in pea and wheat rhizosphere soil, and then ^13^CO_2_ was used to follow the flow of plant-derived carbon into rhizosphere methylotrophs.

## Results and discussion

### Identification of methanol utilisers in rhizosphere soil

Naturally grassed and unfertilised soil from Church Farm (a John Innes Centre site in Norfolk, UK) was used as the basis for this study. The soil from Church Farm was used to produce three experimental soils that were then analysed; pea and wheat plants were grown in containers in the laboratory, and the rhizosphere soils were collected at the reproductive stage of the life cycle of the pea and wheat plants, 4 weeks after planting, and compared with similarly treated but unplanted soil. DNA was extracted from the soils, and the microbial communities were analysed by 16S rRNA gene and methanol dehydrogenase gene PCR amplicon sequencing, using either high-throughput methods (16S rRNA gene, *mxaF*, *xoxF1*, *xoxF2*, and *xoxF5*) or clone library analysis (*mdh2*, *xoxF*3).

Sequencing of 16S rRNA genes from each habitat revealed that the most abundant phylum within the unplanted soil and the rhizosphere soils was the *Proteobacteria* (33%), with *Hyphomicrobiaceae* (13%) being the most abundant family within this phylum (Additional File 1). *Actinobacteria* and *Planctomycetes* were also abundant phyla (approximately 22 and 19% relative abundance) within these soil environments. The 16S rRNA gene sequencing identified genera that contain species previously known to oxidise and grow on methanol (extant methylotrophs) or that contain species that possess methanol dehydrogenase genes (putative methylotrophs) (Additional File 2). Thirty-four methylotrophic genera were identified in the 16S rRNA gene profile of the unplanted soil, at a combined relative abundance of 15.4%, and 35 methylotrophic genera were distributed across pea and wheat rhizosphere soils, at 15.8% and 14.4% relative abundance respectively. The most abundant confirmed methylotrophic genera (*Hyphomicrobium*, *Methylophilus* and *Verrucomicrobium*) and putative methylotrophic genera (*Flavobacterium* and *Bradyrhizobium*) were found in all three habitats.

### Amplification of mdh2 methanol dehydrogenase genes

**Fig. 1:**
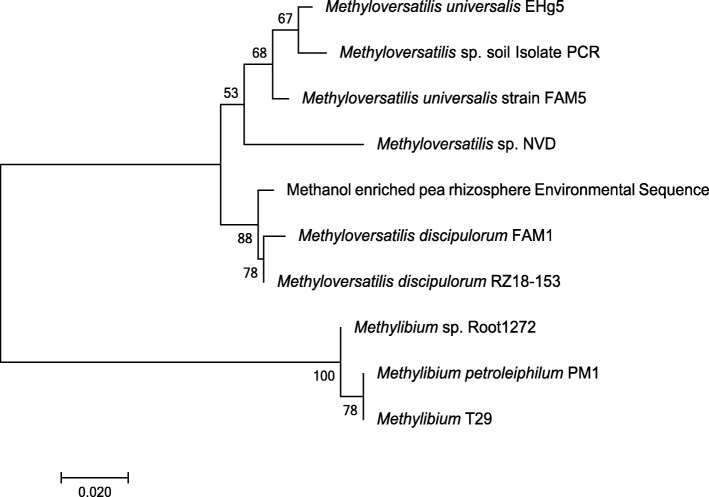
Phylogeny of *mdh2* sequences retrieved from pea plant rhizosphere compared to *mdh2* from methylotrophic bacteria. Sequenced amplicons obtained from environmental DNA and DNA from cultures of methylotrophic bacteria are labelled as “Environmental Sequence” and “Isolate PCR” respectively. Full reference gene sequences were selected from the NCBI nucleotide database. The tree was drawn in Mega7 [30] using the neighbour-joining method. Scale bar indicates 0.02 substitutions per site. Only bootstrap values ≥ 50% (based on 500 replicates) are labelled at branch points. There were a total of 164 amino acid residues in the final dataset

PCR primers for specific amplification of *mdh2* genes were designed by aligning *mdh2* gene sequences from the sequenced genomes of strains of *Methylibium* and *Methyloversatilis* as described in the “Methods” section. The specificity of the primers was assessed by performing PCR using DNA from *mdh2*-possessing bacteria, *Methylibium* sp. ROOT1272 and *Methyloversatilis* sp. LF1. Sequencing confirmed the identity of PCR products as *mdh2* methanol dehydrogenase genes. DNA from unplanted soil and pea rhizosphere soil did not yield PCR amplicons when assayed with the *mdh2* primers. However, when enriched with methanol (see the “DNA-SIP with 13C methanol” section), DNA extracted from pea rhizosphere soil yielded a PCR amplicon, and restriction fragment length polymorphism (RFLP) screening of the resultant clone library identified a single operational taxonomic unit (OTU), with a high degree of identity (96–99% nucleotide identity) with *mdh2* sequences from *Methyloversatilis* (Fig. 1). The absence of *mdh2* products in PCR assays with DNA from the soils that were not enriched with methanol, and the lack of sequence diversity in DNA from the methanol-enriched pea rhizosphere suggests that *mdh2* is not abundant in this environment, although it may be more relevant to other environments, such as in freshwater systems [31], where genera that possess *mdh2*, including *Methyloversatilis*, are more abundant. Alternatively, the low number of *bona fide mdh2* sequences used to design primers may have resulted in primers that are specific to a narrow group of organisms. The identification of additional *mdh2*-possessing organisms might enable the design of primers with broader specificity.

### Diversity of *mxaF* and *xoxF* in soils

The diversity of *mxaF* and *xoxF* genes in the unplanted soil and rhizosphere samples was analysed by amplicon sequencing of PCR products generated using primers developed previously [28]. The *mxaF* amplicons produced from DNA extracted from the unplanted and pea rhizosphere soil were dominated (> 99%) by three OTUs affiliated with *Hyphomicrobium*. This genus was present at 4.5–6% relative abundance in the 16S rRNA gene profile of the unplanted soil and pea rhizosphere bacterial communities, and, of the genera predicted to contain the *mxaF* gene, *Hyphomicrobium* was the most abundant within these environments. The remainder of the *mxaF* sequences (< 1%) clustered with *mxaF* sequences from members of the family *Methylophilaceae* (Additional File 3). The *mxaF* diversity detected in the Church Farm (CF) soil shows similarities to profiles originating from topsoils of other grassland sites reported in a previous study [18]. These authors identified OTUs affiliated with *Hyphomicrobium* and *Methylophilaceae*, which were also detected here in the Church Farm soil. However, we did not detect *mxaF* amplicons affiliated with *Methylobacterium*, which were detected at high abundance in these previously characterised grassland soils from the previous study [18].

**Fig. 2:**
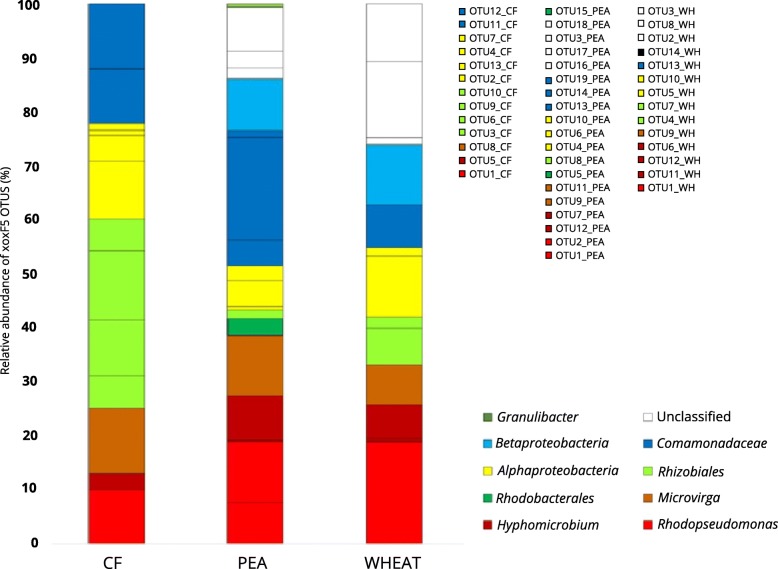
Relative abundance and diversity of *xoxF5* genes in soils. Relative abundance of *xoxF5* amplicons generated from unplanted soil (CF), pea rhizosphere soil (PEA) and wheat rhizosphere soil (WHEAT) as revealed by amplicon sequencing. Abundance of taxonomic groups is shown at the highest level of classification for each OTU, with taxonomy of OTUs inferred from the clustering shown in the phylogenetic tree (shown in Additional File 4)

PCR assays with primers specific for the *xoxF5* clade revealed that the abundant *xoxF5* OTUs retrieved from the unplanted and pea and wheat rhizosphere soils had high similarity to *xoxF5* from members of the *Alphaproteobacteria* and *Betaproteobacteria* (Fig. 2, Additional File 4), principally members of the genera *Hyphomicrobium*, *Microvirga* and *Rhodopseudomona*s. These genera were also detected in the 16S rRNA gene profiles and contain species capable of methanol oxidation [32–34]. The *xoxF5* profiles of the rhizosphere environments were both enriched in OTUs with high similarity to sequences from *Rhodopseudomonas*, *Hyphomicrobium* and the *Betaproteobacteria*. The wheat rhizosphere also contained a greater number of divergent *xoxF* OTUs that could not be assigned a phylogeny. OTUs of *xoxF5* representatives of members of the *Comamonadaceae* were detected in DNA from the pea rhizosphere at relatively high abundance (~ 25%) (Fig. 2)*.*

PCR primers specific to *xoxF4* consistently failed to yield *xoxF4* amplicons with DNA extracted from the unplanted soil, pea rhizosphere soil and wheat rhizosphere soil, indicating that this gene was either absent or below the limit of detection. Libraries of *xoxF1*, *xoxF2* and *xoxF3* amplicons were generated from DNA extracted from unplanted soil. Three of the *xoxF1* OTUs (OTU1, 2 and 3) had high similarity to members of the *Rhizobiales* (*Oharaeibacter*, *Methyloceanibacter* and *Hyphomicrobium*). There was also an OTU (16%, OTU 4) that did not have high similarity to any of the *xoxF1* reference sequences (Additional File 5). The *xoxF3* clone library was dominated by OTU A (26/47 clones), most closely related to *xoxF3* of *Methylobacterium nodulans* (Additional File 6)*.* The additional diversity (OTUs D, F, G, H, I) (Additional File 6) was comprised of sequences clustering with *xoxF3* genes of species of *Methylosinus*, a methanotroph, and *Azospirillum* (relative abundance 25.5% and 14.9% respectively). The most abundant *xoxF2* OTU retrieved by *xoxF2* amplicon sequencing was identical to a *xoxF2* clone obtained after screening a small clone library (see the “Methods” section). Because this *xoxF2* sequence was greater in length than *xoxF2* sequences obtained using Illumina technology, it was used in further phylogenetic analysis. The *xoxF2* sequence did not cluster with any of the reference sequences and showed highest similarity (84%) to a putative *xoxF2* sequence found in metagenome-assembled genomes from studies investigating the microbial diversity of the Chinese and Japanese seas [35, 36] (Additional File 7). These genomes are assigned to the phylum *Candidatus* Entotheonella, which was not detected in the 16S rRNA gene sequence profile of the Church Farm soil.

### Quantification of *mxaF* and *xoxF5* genes in soils using qPCR

The abundance of the *mxaF* and *xoxF5* genes in the unplanted soil and pea and wheat rhizosphere was determined using qPCR. *xoxF5* was selected from the *xoxF* clades for quantification since *xoxF5* has been proven to be a *bona fide* methanol dehydrogenase-encoding gene in multiple species [37–39] and sequencing of *xoxF5* gene amplicons from the three different habitats identified shifts in diversity that were potentially correlated with shifts in abundance. Furthermore, the primers for *xoxF3* and *xoxF1* are unsuitable for qPCR analysis and their redesign was outside of the scope of this work. *xoxF4* was not selected for quantification as it was not detected in the unplanted soil DNA and the primers for *xoxF4* have cross-specificity for *xoxF5* genes in the absence of *xoxF4* [28].

Normalised to 16S rRNA gene copy number, the qPCR assays showed that within the three soil environments tested, *xoxF5* genes were 36–42 times more abundant than *mxaF* genes (Additional File 8). However, the overall abundance of methanol dehydrogenase genes did not differ significantly between the three soil environments (two-way ANOVA, *p* = 0.567). Despite the prevalence of multiple *xoxF5* copies in genomes, these data support the hypothesis that Xox enzymes are more abundant and have a wider distribution than Mxa-type MDHs in this type of environment [24]. This confirms the need to also investigate the diversity and distribution of *xoxF* genes when characterising the diversity of methylotrophic bacteria in environmental studies.

### The impact of plants on soil methylotrophs as assessed by DNA-SIP

Using the same three soil treatments described above (unplanted, pea and wheat rhizosphere), the influence of plant growth on soil methylotrophs was examined to determine the differential response of these plant root-associated soil communities to addition of methanol. DNA-SIP enrichments were set up with each soil type using either ^13^C-labelled or ^12^C-unlabelled methanol. Following incorporation of ^13^C-label, the active methanol-assimilating taxa present in the rhizosphere of these two plant types were compared to the unplanted control soil, by amplicon sequencing of 16S rRNA genes of the ^13^C-labelled DNA retrieved from incubations with ^13^C-methanol after 6 and 17 days of incubation (T1 and T2 respectively).

**Fig. 3:**
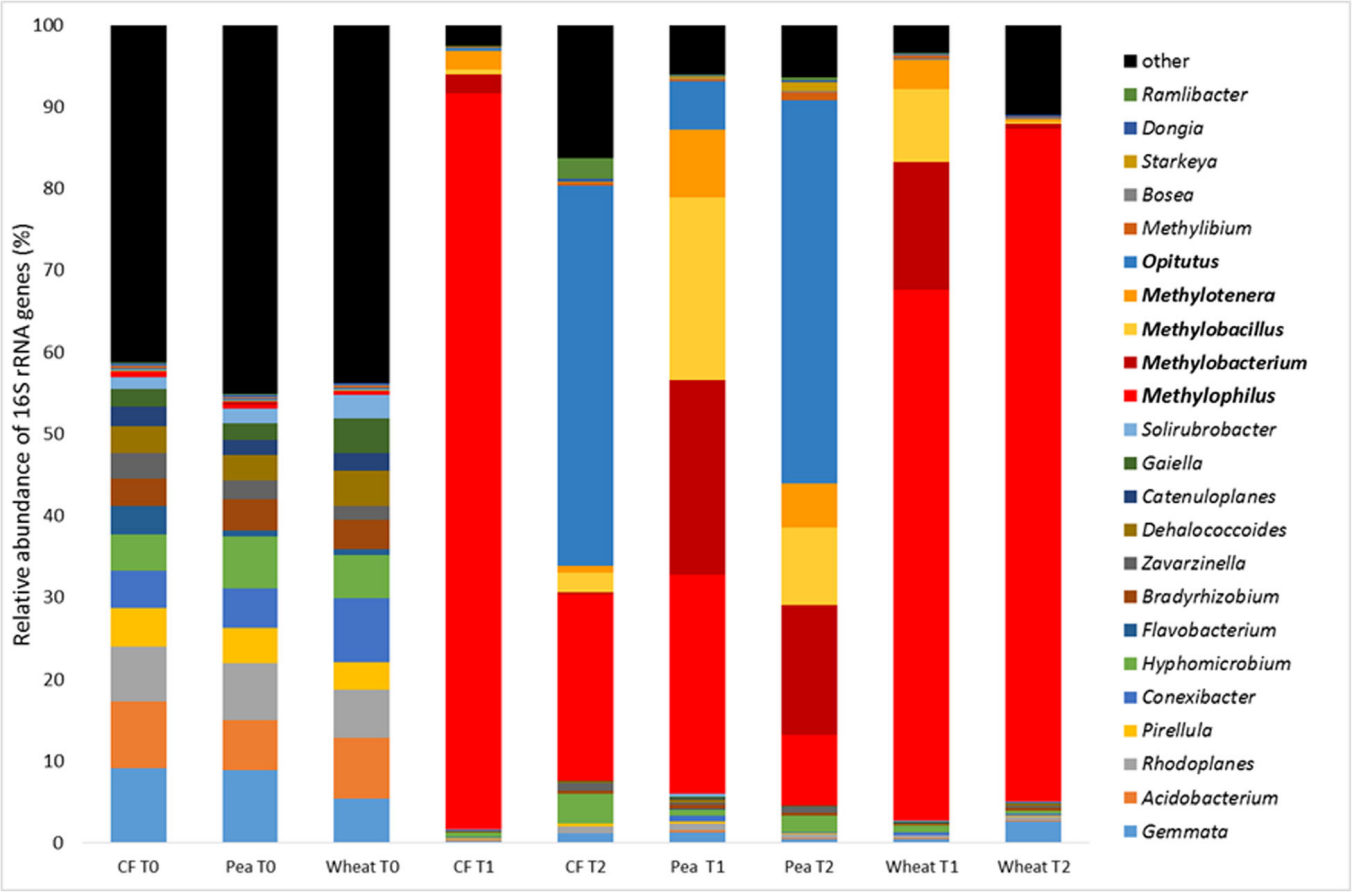
Comparison of the active methylotrophic communities of methanol-incubated unplanted and rhizosphere soils and unamended soil. Relative abundance of taxa based on 16S rRNA gene amplicons produced from DNA extracted from unplanted (CF), pea rhizosphere (Pea) and wheat rhizosphere (Wheat) soil samples at time point zero (T0) and the ^13^C-enriched (heavy) DNA fractions from incubations with ^13^C-methanol for 6 days (T1) and 17 days (T2) are shown. ^12^C controls are detailed in Additional File 10

Analysis of the 16S rRNA genes, after DNA-SIP incubations of the methanol-enriched unplanted soil at T1, identified *Methylophilus* and *Methylotenera* as ^13^C labelled, with *Methylophilus* representing 90% of the 16S rRNA genes retrieved from the heavy DNA. The ^13^C-labelled communities of the T1 heavy fractions of the methanol-enriched pea and wheat rhizosphere soils were more diverse than that of the unplanted soil (Additional File 9). The ^13^C-labelled genera in the pea rhizosphere at T1 were identified as *Methylophilus*, *Methylobacterium*, *Methylobacillus*, *Methylotenera* and *Opitutus* (Fig. 3, Additional File 10), and the same methylotrophic genera were labelled in the wheat rhizosphere, but with a higher relative abundance of *Methylophilus*.

In the unplanted soil incubations, the relative abundance of 16S rRNA genes of *Methylobacillus*, *Methylocystis* and *Methylotenera* increased between T1 and T2, but in addition to these known methylotrophs, *Opitutus* was detected as enriched in the heavy fraction, present at 42% relative abundance 16S rRNA genes retrieved from the heavy fraction. In the pea rhizosphere, of the genera ^13^C labelled at T1, only *Opitutus* increased in relative abundance at T2, increasing from 5 to 24%. However, 13 additional genera were labelled, notably *Starkeya* (1.1%). In the wheat rhizosphere, fewer genera were labelled at T2 compared with T1 and comprised of only *Methylophilus* and *Methylotenera*. The relative abundance of *Methylotenera* decreased tenfold to 0.34%, whereas the relative abundance of *Methylophilus* increased from 64 to 82%.

Additional groups of bacteria ^13^C labelled in the rhizosphere soil DNA-SIP experiment, albeit in low abundance, were *Stigmatella* (0.32% in the wheat rhizosphere and 1.19% in the pea rhizosphere) and members of the phylum *Lentisphaerae* (0.11% in the wheat rhizosphere). Based on the lack of MDH genes in published genome sequences and previous phenotypic characterisation of representatives of *Stigmatella* and *Lentisphaerae* [40, 41], it is possible that they were ^13^C labelled by cross feeding. Enrichment of *Opitutus* was also unexpected, as members of this genus are not known to be methanol-oxidising bacteria [42].

### Metagenomes reconstructed from DNA-SIP experiments

**Fig. 4:**
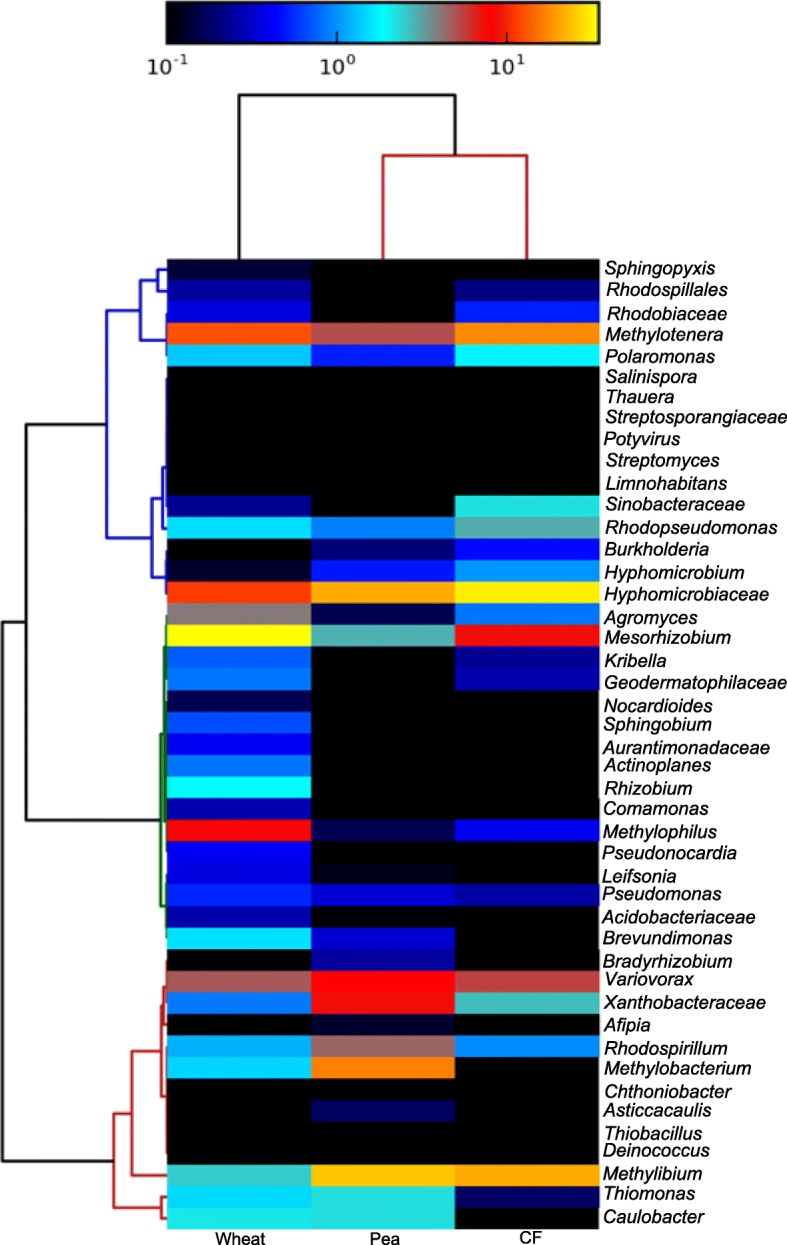
Metagenome-derived community profiles produced from the ^13^C-enriched (heavy) DNA fractions of ^13^C methanol-enriched soils. Sequences were revealed by shotgun sequencing of ^13^C-enriched DNA extracted from wheat rhizosphere soil (Wheat), pea plant rhizosphere soil (Pea) and unplanted soil (CF) incubated with ^13^C-labelled methanol for 17 days. Relative abundance of taxa is indicated by the colorimetric key assigned with a log scale, with yellow indicating abundance > 10^1^ cells of a taxon and black representing an absent taxon

In addition to sequencing 16S rRNA gene amplicons, the active methanol-assimilating taxa in the rhizosphere and unplanted soils were further characterised by shotgun sequencing the ^13^C-labelled DNA retrieved from the DNA-SIP incubations enriched with ^13^C-methanol at T2, resulting in a metagenome for each of the three environments. Metaphlan2 (2.0) [43] was used to analyse the taxonomic composition of the three metagenomes using the presence of taxonomically informative marker genes (the database of these marker genes can be accessed at http://huttenhower.sph.harvard.edu/metaphlan). The metagenomes were dominated by bacteria, specifically Proteobacteria. Gene sequences identified as from *Eukarya*, *Archaea* or viruses were present at below 0.1% relative abundance (Fig. 4).

Consistent with the 16S rRNA gene profiling described above, this metagenomics approach identified members of the *Methylophilaceae* as highly abundant in all three environments. Furthermore, a key genus delineating the methanol-enriched plant environments from the methanol-enriched unplanted soil was *Methylobacterium.* This was present at 14.7% relative abundance in the pea rhizosphere and 1.5% in the wheat rhizosphere. It was not detected in the unplanted controls. Analysis of the metagenomes confirmed the differences observed between the planted and unplanted soils using 16S rRNA gene amplicon sequencing. The metagenomes revealed a higher diversity than the 16S rRNA gene amplicon approach (Additional File 9). The additional genera detected in the metagenomes that were shared between the environments include *Mesorhizobium*, *Methylibium*, *Variovorax* and *Rhodospirillum*. The metagenomes also showed more differences in community composition between the two planted soils, with *Comamonas*, *Sphingobium*, *Rhizobium*, *Leifsonia*, *Mesorhizobium* and *Methylophilus* all being present at greater abundance in DNA from the methanol-enriched wheat community whilst *Variovorax*, *Bradyrhizobium*, *Afipia*, *Asticcacaulis* and *Rhodospirillum* were present at greater abundance in DNA from the methanol-enriched pea rhizosphere community. In comparison with the unplanted soil, these data show that the rhizosphere soils contain multiple taxa poised to take advantage of additions of methanol. This lends weight to the hypothesis that plants support a higher diversity of bacteria, in particular methylotrophs, than are present in unplanted soil.

The metagenomes were screened for genes of interest using the blast function of BioEdit. This identified the presence of genes encoding for methanol dehydrogenases (*xoxF* and *mxaF*), enzymes involved in methylated amine utilisation (*tmmD*, *dmmD*, *mauA* and the N-methylglutamate pathway) and formaldehyde and formate oxidation in all three metagenomes. The screening also identified all the genes of the ribulose monophosphate and serine cycles. Genes encoding the complete pathways for assimilatory sulfate reduction, denitrification and nitrogen fixation were also detected in the three metagenomes, providing an insight into the energy and nitrogen yielding pathways active in the three soil habitats. The screening for genes of interest did not reveal a difference in the presence of these metabolic pathways between the metagenomes. To investigate differences in the abundance and diversity of methanol dehydrogenase-encoding genes in the three methanol-enriched soil habitats, the assembled and unassembled reads of the metagenomes were screened with representative sequences of each clade of methanol dehydrogenase-encoding gene (*xoxF1*, *xoxF2*, *xoxF3*, *xoxF4*, *xoxF5*, *mxaF* and *mdh2*). The abundance of methanol dehydrogenase genes differed between the three metagenomes (Additional Files 11, 12, 13, 14, 15, 16). *mxaF* and *xoxF4* were present at higher abundance in the two plant habitats relative to the unplanted soil, whereas *xoxF5* and *xoxF3* were more abundant in the pea rhizosphere relative to the unplanted soil and wheat rhizosphere. *mdh2* sequences were only detected in the methanol-enriched pea rhizosphere and unplanted soils, and these sequences showed high (> 82%) sequence identity to those of *Methylibium* and *Methyloversatilis* (Additional File 16). The diversity of the *xoxF3* sequences detected in the metagenomes was greatest in the methanol-enriched pea rhizosphere soils (Additional File 16), which included sequences with high sequence identity to those possessed by members of the genera *Methylobacterium* and *Mesorhizobium*, in addition to the *Comamonadaceae* sequences detected in the other two enriched soil habitats (Additional File 16). *xoxF2* and *xoxF* outgroup sequences (the *Acidiphilum* and *Methylosinus trichosporium* sequences that do not cluster with any established clade [23]) were not detected in the three metagenomes.

*xoxF5* was the most abundant methanol dehydrogenase-encoding gene in the pea rhizosphere and unplanted soil but was only the second most abundant in the wheat rhizosphere, where the most abundant was *xoxF4*. Compared to the number of copies of *mxaF* and *xoxF5* determined by qPCR for soils that were not enriched with methanol, these data show that following enrichment with methanol, *xoxF5* was present at greater abundance than *mxaF*, but both genes were present at the same order of magnitude in all three soil habitats. The *xoxF1*, *xoxF5* and *mxaF* sequences detected in the metagenomes arising from the methanol-enriched pea and wheat rhizospheres and unplanted soils showed similar patterns of diversity to the sequenced amplicons produced from the unenriched soil. The *xoxF1* sequence profiles revealed low levels of diversity, with the detected *xoxF1* sequences showing high sequence identity to those identified in genomes of strains of *Hyphomicrobium* and *Methyloceanibacter* (Additional File 12). The diversity of *mxaF* sequences was also low, with the *mxaF* sequences detected having high sequence identity to *mxaF* genes from *Hyphomicrobium*, *Methylobacterium* and *Methylophilus* in all three metagenomes (Additional File 15). The *xoxF5* sequences in the three metagenomes showed high sequence identity to sequences identified in members of the *Alphaproteobacteria* (*Rhizobium*, *Methylobacterium* and *Hyphomicrobium*) and *Betaproteobacteria* (*Methylibium* and *Comamonadaceae*), with similar sequence diversity detected between the three soil habitats (Additional File 14). There was also similarity in the diversity of the *xoxF4* sequences detected in the three methanol-enriched habitats, with high sequence identity to members of four genera within the *Methylophilaceae* (*Methylomonas*, *Methylophilus*, *Methylobacillus* and *Methylovorus*) (Additional File 13), but this might be an artefact of the low diversity of this clade of methanol dehydrogenase-encoding gene.

To further investigate the diversity of the methanol-enriched environments, assembled sequence data from the three metagenomes were binned into metagenome assembled genomes (MAGs). The binning produced 10 MAGs of sufficient quality (completeness score > 70% and contamination < 10%), meeting currently accepted criteria for medium- to high-quality MAGs [44]) (Additional File 17). These MAGs were also screened for genes of interest (Additional File 18). One genome (vs26) was identified as a *Rubrivivax*. This MAG contained a *xoxF5* methanol dehydrogenase-encoding gene, genes encoding a complete tetrahydromethanopterin formaldehyde oxidation pathway [45], thiosulfate oxidation (*soxABXYZ*) and assimilatory sulfate reduction (*cysCDHIJN*), as well as an incomplete serine cycle. The genome binning also produced a MAG classified as a *Methylobacterium* (ss20), an abundant genus in the methanol-enriched pea and wheat rhizosphere soils. This MAG contained *mxaF* and *xoxF5* methanol dehydrogenase genes, and genes encoding the complete N-methyl glutamate pathway for methylamine utilisation [46], an incomplete serine cycle and one of each of the four forms of formate dehydrogenase [47–49]. However, we cannot rule out the possibility that the incomplete serine cycle of these MAGs may be an artefact resulting from imperfect sequence assembly or binning (predicted MAG completeness 97% and 72% respectively).

Of the remaining MAGs, three were members of the order *Methylophilales*, highly enriched in all of the soil environments supplemented with methanol. The methanol dehydrogenase gene of the MAG-designated *Methylotenera* ss03 was of note, as the *xoxF3* did not cluster with those of other *Methylophilaceae* (i.e. *Methylobacillus flagellatus*), but instead with *Variovorax paradoxus* strain S110 and sequences from the Alphaproteobacteria (Additional File 19), suggesting that the diversity of this clade of methanol dehydrogenase within the *Methylophilaceae* is greater than previously detected. The MAG-designated Methylophilales ss01 and ss29 were of interest as they showed high levels of similarity to the strains *Methylobacillus* sp. strain MM3 (97% average nucleotide identity (ANI)) and *Methylovorus* sp. strain MM2 (99% ANI) respectively, both of which were previously isolated from the same environment [50]. Despite the fact that enrichment and isolation techniques may not capture all representatives of the *bona fide* natural community, this suggests that both strains may have been active members of the methanol-oxidising community of this soil. In addition to methanol dehydrogenase genes, these MAGs contain formate dehydrogenases and partially complete ribulose monophosphate cycles.

Further analysis of the MAGs Archaea vs43, *Bdellovibrio* vs70, *Deltaproteobacteria* ss68 and *Verrucomicrobia* vs53 and ss71 showed that none of them contained a methanol dehydrogenase-encoding gene or genes encoding formaldehyde utilisation pathways. However, the MAGs *Verrucomicrobia* ss101 and *Deltaproteobacteria* ss68 both contain copies of a formate dehydrogenase (*fdh4*)-encoding gene and the genome ss68 possessed genes encoding dimethylamine and trimethylamine dehydrogenases, implying that these strains of bacteria may be able to utilise some C1 compounds as carbon and/or nitrogen sources. The enrichment of these taxa with ^13^C could therefore be explained by the utilisation of exuded C1 compounds (i.e. formate), utilisation of other exuded organic compounds, the fixation of ^13^C-labelled CO_2_ produced by the methanol oxidising methylotrophs (in the case of vs43, which contains the gene for ribulose-1,5-bisphosphatecarboxylase/oxygenase) or, in the case of *Bdellovibrio* (vs70), predation on the methanol oxidising methylotrophs [51].

### Phylogenetic analysis of the exudate-utilising community of the pea rhizosphere

In a third series of experiments, DNA-SIP was also utilised to investigate whether methylotrophic bacteria were utilising carbon exuded from the roots of plants. Pea plants were incubated in a ^13^CO_2_ or ^12^CO_2_ (control) atmosphere for 12 days to allow sufficient ^13^C label to be incorporated into plant biomass and then for ^13^C-labelled plant exudate released from roots to be assimilated by microbes in the rhizosphere. Two CO_2_ concentrations were used, 350 and 1000 ppmv, reflecting environmental and elevated levels. Three hundred fifty parts per million volume was selected as the concentration of CO_2_ to supply to the environmental test group, as CO_2_ was also released by the soil, and this ensured the concentration did not exceed environmental levels (420 ppm).

**Fig. 5:**
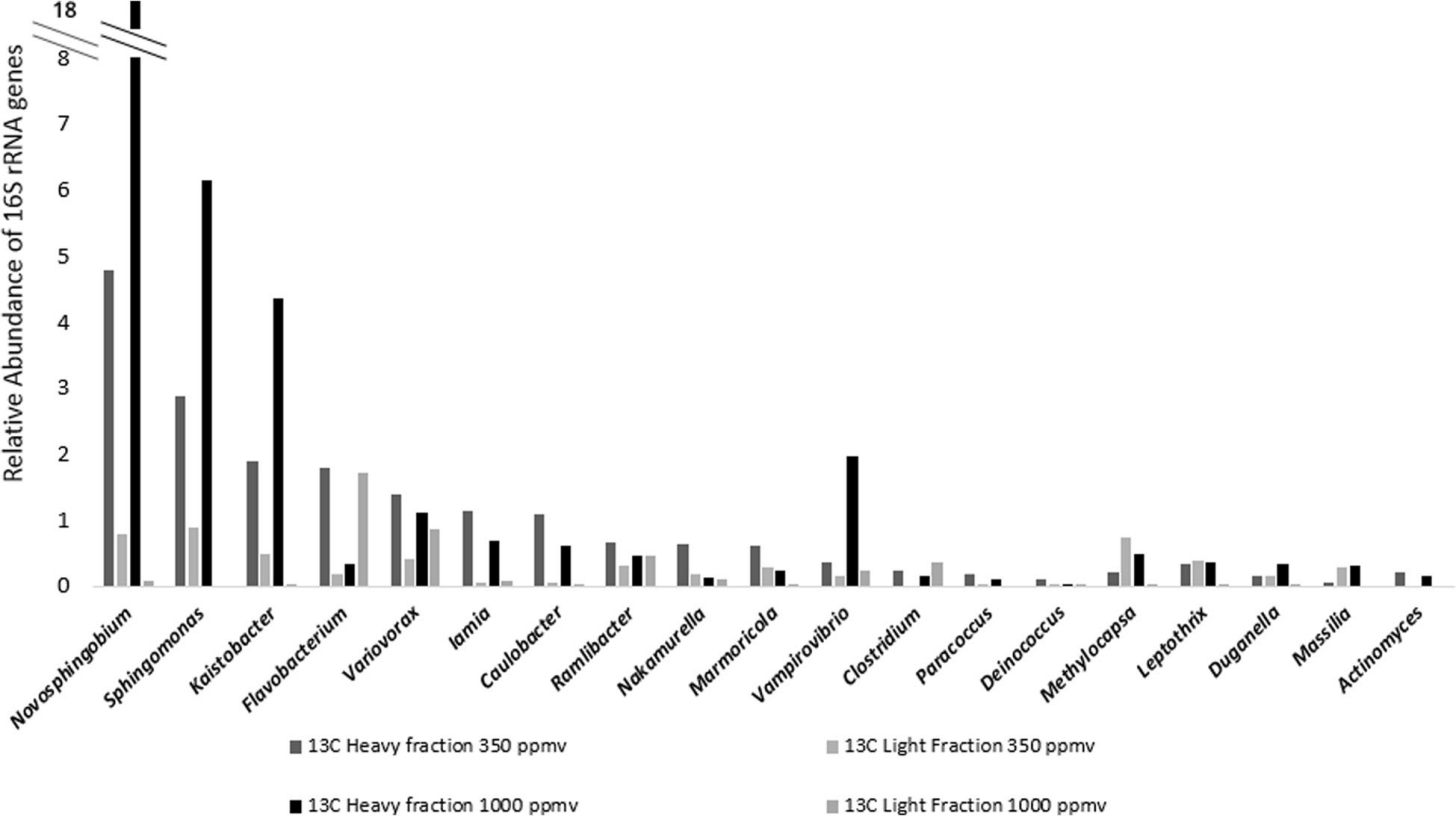
Relative abundance of exudate-utilising genera in the rhizosphere of pea plants. Relative abundance of taxa identified as ^13^C labelled in the ^13^C test group from the heavy fractions and light fractions of DNA extracted from the rhizospheres of pea plants incubated with ^13^C-carbon dioxide at ambient (350 ppm) and elevated (1000 ppm) concentrations for 12 days. Genera that were also ^13^C labelled in the unplanted test group were excluded and are detailed in Additional Files 26 and 27. The data show the mean of duplicate incubations

The bacteria in the pea rhizosphere that were active utilisers of plant exudates were identified by sequencing of 16S rRNA gene amplicons generated from heavy and light DNA fractions retrieved from rhizosphere and unplanted (control) soils. Analysis of 16S rRNA gene sequences retrieved from heavy fractions of DNA obtained in DNA-SIP experiments with pea plants incubated with ^13^CO_2_ indicated that labelling of bacteria of the genera *Novosphingobium* (4.8% relative abundance after enrichment with 350 ppmv ^13^CO_2_ and 17.7% relative abundance after enrichment with 1000 ppmv), *Kaistobacter* (1.9% at 350 ppmv and 4.4% at 1000 ppmv), *Sphingomonas* (2.9% at 350 ppmv and 6.2% at 1000 ppmv), *Paracoccus* (0.2% at 350 ppmv), *Variovorax* (1.4% at 350 ppmv), *Flavobacterium* (1.8% at 350 ppmv) and *Ramlibacter* (0.% at 350 ppmv), *Methylocapsa* (0.5% at 1000 ppmv) and *Leptothrix* (0.4% at 1000 ppmv) occurred (Fig. 5). With the exception of *Novosphingobium* and *Kaistobacter*, these genera contain species that are either methylotrophs or whose genomes contain *xoxF* genes [11, 52–54]. The addition of an elevated concentration of carbon dioxide to growing grasses and sedges has previously been shown to impact on the rhizosphere community [55]. The observation that growing pea plants with different levels of CO_2_ might favour the growth on root exudates of different groups of methylotrophs is interesting and warrants further investigation in the future.

*Variovorax*, *Ramlibacter* and *Leptothrix* genera within the *Comamonadaceae* were ^13^C labelled in the rhizospheres of pea plants incubated with ambient and elevated ^13^CO_2_. Strains from these genera have previously been found in rhizosphere environments [56–59]*.* The family *Comamonadaceae* contains bacteria that are metabolically versatile and can use a broad range of carbon substrates, enhance the cycling of sulfur in soil and suppress fungal pathogens [57, 60]. Genera within this family include *Variovorax* and *Delftia*, which contain species known to grow on methanol [58, 61]. An examination of all available genomes of members of the *Comamonadaceae* revealed that representatives of 28 out of 34 genera contain *xoxF* genes. With the recent discovery of the role of lanthanides in methylotrophy, there is a clear need to retest representatives of the *Comamonadaceae* for their ability to grow on methanol in medium supplemented with these rare earth elements.

Labelling of members of the *Sphingomonadaceae* and *Actinobacteria* in our DNA-SIP experiments is consistent with previous reports with other plant species [62–64], and the utilisation of exudates by members of the *Sphingomonadaceae* has been observed in stable isotope probing experiments and the rhizosphere of rice plants [65]. *Actinobacteria* are proposed to play a role as plant growth promoting bacteria through the production of antimicrobial or antifungal agents, plant hormones and siderophores [87 and references therein]. In ^13^CO_2_ rhizosphere SIP studies with oil seed rape, wheat, maize and *Medicago truncatula*, *Actinobacteria* were also observed to use root exudates [2, 64]. The ^13^C labelling of members of the *Actinobacteria* and *Sphingomonadaceae* in stable isotope probing studies implies they incorporated carbon exuded by the plant, suggesting that they were enriched in the rhizospheres of different plant species because of direct utilisation of plant exudates rather than cross feeding.

Comparing the 16S rRNA gene and methanol dehydrogenase gene amplicons produced from DNA extracted from the unenriched unplanted soil and rhizosphere soils to the taxonomic profiling of the labelled communities from soils that were enriched with either ^13^C-methanol or ^13^CO_2_ identified that a small portion of diversity was common between these different test groups (e.g. *Variovorax*, Additional Files 20, 21, 22). However, beta diversity analysis (Additional File 23) clearly shows that the communities in the methanol-enriched samples cluster separately from the CO_2_-enriched samples. This difference is largely due to the high levels of enrichment of members of the *Methylophilaceae* in the soils amended with methanol and their absence in the exudate utilising population of the pea rhizosphere.

## Conclusions

Growing plants are a major source of methanol, and the microbial community phyllosphere of multiple plant species has been shown to include methylotrophs that are highly abundant [66–68]. However, there have been few studies attempting to characterise the impact of plant growth on the diversity and activity of methylotrophs in the rhizosphere and whether they are using carbon directly from the plant. In this study, methylotrophs were shown to be abundant in the unplanted soil, pea rhizosphere soil and wheat rhizosphere soil, and their diversity was influenced by pea and wheat plants. The use of *xoxF* and *mxaF* as functional gene markers revealed a greater diversity of methylotrophs in the rhizosphere than previously observed by 16S rRNA gene sequencing, with *xoxF* shown to be the more relevant indicator of methylotrophy in these soil environments and also an order of magnitude more abundant than *mxaF*. However, *mdh2* currently is of limited utility in characterising the diversity of methylotrophs in soils.

Metagenome sequencing of ^13^C-labelled DNA extracted from pea and wheat rhizosphere soils and unplanted soils enriched with methanol confirmed that the growth of pea and wheat plants influenced methylotrophy. Interestingly, both plant associated environments showed the same shift in the microbial community profile, revealing a greater diversity of members of the *Methylophilaceae* and *Methylobacterium*, a cosmopolitan genus possessing plant growth-promoting traits and commonly associated with plants [30, 69, 70]. ^13^CO_2_ labelling of growing pea plants also confirmed that methylotrophs present in the pea rhizosphere were actively utilising carbon exudate from the plant. Comparing the methylotrophic genera detected in the pea rhizosphere, by sequencing of methanol dehydrogenase genes from the unplanted soil, from the exudate-utilising population from the ^13^CO_2_ SIP experiment and from soils enriched with ^13^C methanol, revealed that, using these approaches, only a minority of the diversity was shared. The differential enrichment of methylotrophs between the two SIP experiments indicates that there could be selection for some genera of methylotrophs in response to the higher concentrations of methanol (e.g. *Methylophilaceae*) whilst others can utilise methanol at a wider range of concentrations (e.g. *Comamonadaceae*).

Plants can also influence the availability of micronutrients, soil structure, pH and redox potential [62], and these factors could play a role in the recruitment of methylotrophs to the rhizosphere. Furthermore, the specific growth stages of the plants used in this series of experiments and their stress state might affect the amount and nature of exudate released from the roots [19, 71]. Both factors might impact on the activity of methylotrophs in the soil. Additional studies are therefore needed to define the exact relationship between methylotrophs and the rhizosphere.

## Methods

### Chemicals and reagents

Analytical grade reagents used were from Sigma-Aldrich (MO, USA), Melford Laboratories (Ipswich, UK) or Fisher Scientific (Loughborough, UK). Molecular biology grade reagents were from Thermo Fisher (MA, USA), Promega UK (Southampton, UK), Qiagen (Germany) and Roche (Switzerland). Gases were supplied by BOC (UK). ^13^CO_2_ and ^13^C-labelled methanol were supplied by Cambridge Isotope Laboratories (MA, USA). All ultracentrifuge work involved using tubes, rotors and ultracentrifuges from Beckman Coulter (CA, USA).

The experimental workflow for the research described in this study is detailed in Additional File 24.

### Collection, processing and storage of soil

Soil was collected in April 2015 from a naturally grassed and unfertilised part of John Innes Centre Church Farm (Norfolk, UK) (52.6276 N, 1.1786 E). The top 10 cm was removed from a 1 m^2^ section and then soil to 20 cm depth was removed, air dried and sieved through 10 mm and 5 mm sieves which removed stones, roots and other detritus. This soil (designated “bulk soil”) was then used in all experiments.

### Extraction of nucleic acids from soil

DNA was extracted from soil samples using a cetyltrimethyl ammonium bromide (CTAB)-based method [72] and quantified using Qubit fluorometric quantitation (Thermo Fisher).

### Germination and growth of plants

Paragon wheat seeds (*Triticum aestivum* var. Paragon) were sterilised by washing the seeds in 5% (v/v) sodium hypochlorite solution for 1 min. Pea seeds (*Pisum sativum* var. Avolar) were sterilised by washing the seeds in 95% (v/v) ethanol for 1 min, washing with sterile H_2_O and soaking in 2% (w/v) sodium hypochlorite for 5 min. Pea and wheat seeds were then washed in sterile H_2_O and placed in a petri dish on filter paper disks moistened with sterile H_2_O [13]. The seeds were left in the dark for 3 days to germinate and manually inspected for fungal contamination before planting in 10 cm × 10 cm pots in bulk soil and growing at 22 °C under long day regimes (16:8 h) in plant growth rooms. Pots with unplanted soil were incubated alongside the growing plants as unplanted controls. Plants were harvested after 4 weeks of growth. Excess soil was removed by shaking the roots three times. Soil that remained attached to the roots after shaking was defined as rhizosphere soil. Rhizosphere soil was removed by transferring the roots to Falcon tubes, submerging in phosphate buffered saline (PBS) and vortexing for 30 s. Tubes were centrifuged at 3200×*g* for 15 min to pellet soil. Root material was removed and the supernatant discarded. DNA was extracted from three separate 0.5 g aliquots of soil and subsequently pooled to produce a composite sample.

### DNA-SIP with ^13^C methanol

Two grams of aliquots of rhizosphere soil, collected as described above, and unplanted soil were dispensed into 120-ml serum vials. Forty millilitres of sterile H_2_O was added to reduce the heterogeneity of the soil sample within the serum vials and to facilitate substrate distribution. Vials were then supplemented with ^13^C methanol or ^12^C methanol to a concentration of 250 μM and sealed. Each test group was performed in triplicate. The serum vials were incubated at 30 °C without light in a shaking incubator (120 rpm). The concentration of methanol in the headspace of the serum vials was measured using gas chromatography (GC) on an Agilent 7820A instrument, using a flame ionisation detector, a Porapak Q column (6 ft × 1/8″ ×2.1 mm) and helium carrier gas (injector temperature, 300 °C; detector temperature, 300 °C; oven temperature, 115 °C). After depletion of methanol, samples were resupplied with methanol to the same concentration. Serum vials were opened every second day and flushed with air to prevent the development of anaerobic conditions and to avoid the build-up of ^13^CO_2_ within vials. After 6 days, when methanol oxidation stopped, samples were taken from enrichments for DNA extraction (time point 1). Dilute nitrate mineral salts medium [73] (1 ml) was supplied to the serum vials on day 7 to establish whether the enrichments were nutrient limited and the incubations were continued. Methanol consumption resumed. After 17 days, a total of 200 μmol of ^13^C had been consumed in incubations and soil was collected for DNA extraction (time point 2). DNA was extracted from all soil samples, and caesium chloride density gradient centrifugation was used to separate the ^13^C- and ^12^C-labelled DNA from 1–3 μg of DNA from each test group according to established protocols [74]. During fractionation of CsCl gradients, twelve samples were collected and the density of CsCl in each fraction estimated by measuring the refractive index (Reichert AR200). DNA was then recovered by precipitation [74].

### DNA stable isotope probing with ^13^CO_2_

Pea plants were grown in 10 cm × 10 cm pots in bulk soil under long day growth conditions (16 h:8 h) for 16 days. After 16 days, eight pea plants and eight unplanted soil controls were transferred to acrylic tubes (approx. 3.8 L volume, 400 mm height × 110 mm internal diameter). All plants and unplanted soil controls were transferred to medium day light conditions (12 h:12 h). The acrylic tubes were flushed with carbon dioxide depleted air, sealed with plastic lids and injected with either ^13^CO_2_ or ^12^CO_2_ to a final concentration of either 350 ppmv or 1000 ppmv, with each experimental condition in duplicate. Carbon dioxide was measured by GC on an Agilent 7890A instrument equipped with a nickel catalyst, using a flame ionisation detector, an HP plot/Q (30 m × 0.530 mm, 40 μM film) and helium carrier gas (injector temperature, 250 °C; detector temperature, 300 °C; oven temperature, 50 °C). Carbon dioxide was replenished to the target concentration every 20 min during the light period. After 12 days, plants were harvested and DNA extracted from the rhizosphere soil. DNA (4 μg) for each sample was processed via ultracentrifugation and fractionation as described above.

### Criteria for confirming ^13^C labelling of DNA from target microbes

Specific criteria were applied when analysing DNA sequence data from both SIP experiments to establish which taxa of bacteria were labelled. In order to be included in the analysis, the relative abundance of a specific taxon in the ^13^C-heavy DNA fraction had to be greater than 0.1%. The criteria that needed to be fulfilled for a particular taxon to be considered ^13^C labelled were as follows: (1) the relative abundance in the ^13^C-heavy fraction should be higher than in the ^12^C-heavy fraction (^13^C_H_ > ^12^C_H_) and (2) the taxon should be enriched in the heavy fraction of ^13^C incubations (i.e. the relative abundance in the heavy fraction should be greater, by a specific factor, than in the light fraction (^13^C_H_ > *k* × ^13^C_L_), but this should not be the case for ^12^C incubations (^12^C_H_ ≤ ^12^C_L_). The factor *k* was chosen as *k* = 10 for the methanol SIP experiment, due to the substrate based stable isotope approach used and the probability of cross feeding, but *k* = 2 in the ^13^CO_2_-labelling SIP experiment, due to the more transient nature of the ^13^C labelling and the lower input of ^13^C. Any enrichment of autotrophic bacteria could be accounted for and observed by incubating unplanted soil controls in a ^13^CO_2_ atmosphere. Any taxon identified as ^13^C labelled in the unplanted test group was excluded from the list of taxa identified as ^13^C labelled in the rhizosphere test group (Additional Files 25 and 26).

### Polymerase chain reaction (PCR) and quantitative PCR assays

Amplification of products by PCR was performed in 25 μL reaction volumes using a BIORAD Tetrad 2 thermal cycler. The reaction mixture was 1× Master Mix (PCR Biosystems, UK), 0.4 μM forward primer and 0.4 μM reverse primer. PCR primers and amplification protocols used to screen for 16S rRNA, *mxaF*, *xoxF1*-*5* and *mdh2* genes are detailed in Additional File 27. PCR products were purified using NucleoSpin Gel and PCR clean-up columns (Macherey-Nagel, Germany) according to the manufacturer’s instructions. The copy number of 16S rRNA, *mxaF* and *xoxF5* genes in DNA and cDNA samples was estimated using quantitative PCR (qPCR) (Applied Biosystems Step one plus real-time PCR system, Thermo Fisher, MA, USA). The reaction mixture was BioLine Sensifast Hi Rox master mix, 0.4 μM each primer and with the addition of bovine serum albumin (0.2 μg). Standards were prepared using *xoxF5* and *mxaF* PCR products amplified from DNA of *Methylocella silvestris* BL2, diluted to a copy number of 10^8^ to 10^1^ per microlitre. Three biological replicates from each environment were tested, each with three technical replicates. The efficiency of the amplification was 98% for *mxaF* and 83% for *xoxF5*. A two-way ANOVA test was performed using the R package dplyr to test for significant differences between the test groups.

### Design of PCR primers to amplify *mdh2*

PCR primers were designed to amplify *mdh2* genes from DNA isolated from soils. The primers were based on conserved regions identified by aligning five *mdh2* sequences (*Methylibium petroleiphilum* PM1 AAEM01000000, *Methyloversatilis universalis* strain FAM5 EU548062, *Methyloversatilis universalis* EHg5 JN808865, *Methyloversatilis discipulorum* strain RZ18-153 EU548066, *Methyloversatilis discipulorum* strain FAM1 EU548063.1) using the MUSCLE algorithm in MEGA7 [75] and screening the alignment for a conserved region of 18-20 nucleotides, allowing for a maximum of three non-conserved bases. Specificity of the *mdh2* primers was tested by performing PCR using DNA extraction from two strains of methylotrophic bacteria, *Methylibium* sp. ROOT1272 (NZ_LMDY00000000) and *Methyloversatilis* sp. soil isolate (MK795690), as positive controls and DNA from strains of bacteria that do not possess *mdh2* as negative controls (*Methylobacillus* sp. MM3 (NZ_LXTQ00000000), *Methylovorus* sp. MM2 (NZ_LXUF00000000), *Hyphomicrobium* sp. MMN) (MK795690). DNA extracted from unplanted soil, pea rhizosphere soil and methanol-enriched pea rhizosphere soil was used as template to generate PCR amplicons of *mdh2*. Mdh2-specific PCR products (~ 500 bp) were cloned using the Promega pGEM-T Easy vector system according to the manufacturer’s instructions. Cloned PCR products were amplified using M13 primers (Additional File 27), and 20 clones from each library were screened by restriction fragment length polymorphism (RFLP). PCR products were digested using the restriction enzymes *Rsa*I and *Alu*I. RFLP profiles were analysed by gel electrophoresis using 2% (w/v) agarose gels, and representative *mdh2* genes from different soil DNA samples were sequenced.

### Sequencing of 16S rRNA and MDH genes

16S rRNA gene amplicons were sequenced using Roche 454 (3000 reads) and Illumina MiSeq (20,000 reads) technology by Molecular Research LP (Shallowater, TX, USA). 16S rRNA gene amplicons produced using the 454 and Illumina platforms were processed by Molecular Research LP through their proprietary pipeline. Sequences were depleted of barcodes and primers then short sequences < 200 bp were removed, together with sequences with ambiguous base calls and sequences with homopolymer runs exceeding 6 bp. Sequences were then denoised, and operational taxonomic units (OTUs) were defined as clustering at 97% similarity, following removal of singleton sequences and chimaeras [76–81]. Final OTUs were taxonomically classified using BLASTn against a curated database derived from GreenGenes, RDPII and NCBI (www.ncbi.nlm.nih.gov, http://rdp.cme.msu.edu) [82]. Beta diversity analysis was performed using the bioinformatics platform Qiime [83] to identify similarities between different the communities profiled by the sequencing of 16S rRNA gene amplicons. Weighted and unweighted UniFrac analysis was performed on 16S rRNA gene amplicons produced from the unenriched, unplanted soils and pea and wheat rhizosphere soils in addition to the heavy and light fractions produced from the DNA-SIP experiments performed with ^13^C-methanol and ^13^CO_2_.

Reads of functional (methanol dehydrogenase) genes sequenced using the 454 platform were analysed using a modified version of a published protocol [28]. SFF files were processed using Mothur [84] to convert the raw files into flowgrams, which were then translated to nucleotide sequences. USEARCH [85] was used for identification and removal of chimeric sequences. Sequences were clustered into OTUs using USEARCH [78], using similarity values of 80%. OTUs were aligned using the MUSCLE algorithm against a database containing representative sequences from different clades of PQQ dehydrogenase. OTUs that clustered with each clade were re-aligned at the amino acid level using a database of sequences specific to that clade (Table 1). Phylogenetic trees were produced in MEGA7 [75] using the neighbour joining algorithm with bootstrap values of 500. 454 sequencing of the *xoxF3* amplicon produced data that was not of sufficient quality, and therefore, a clone library of 100 clones was made from the *xoxF3* PCR amplicon obtained from DNA extracted from unplanted soil. This clone library was constructed and screened via RFLP as described above.

**Table 1:** Number of OTUs in sequenced *mxaF* and *xoxF* amplicons produced from DNA extracted from soil

Gene	Soil environment	Sequencing platform	Number of OTUs
*xoxF1*	Unplanted	Roche 454	4
*xoxF2*	Unplanted	Sanger	1
*xoxF3*	Unplanted	Sanger	6
*xoxF5*	Unplanted	Roche 454	13
*xoxF5*	Pea rhizosphere	Roche 454	19
*xoxF5*	Wheat rhizosphere	Roche 454	14
*mxaF*	Unplanted	Roche 454	4
*mxaF*	Pea rhizosphere	Roche 454	4

Amplicons were produced from DNA samples from unplanted soil, pea rhizosphere soil and wheat rhizosphere soil and analysed by either 454 amplicon sequencing or Sanger sequencing. OTUs were produced using an 80% identity clustering threshold

### Metagenome sequencing and analysis

DNA from the ^13^C-heavy fractions of the methanol SIP experiment was pooled, quantified and sequenced by the Centre for Genomic Research at the University of Liverpool. Sequencing was performed using paired-end sequencing (2 × 150 bp) on an Illumina HiSeq 4000. Short sequences and sequences of poor quality were excluded from the files using the program Trimmomatic (0.36) [86], and the quality of the metagenomes was assessed using QUAST, including the MetaQUAST expansion (5.0.0) (Table 2) [87].

**Table 2:** Statistics of metagenomes assembled from the heavy fraction of methanol-enriched rhizosphere and unplanted soils

	Metagenome		
	Pea	Unplanted	Wheat
# contigs (≥ 0 bp)	1151414	1251579	981758
# contigs (≥ 1000 bp)	195697	192658	106074
Total length (≥ 0 bp)	934363676	922084398	616537133
Total length (≥ 1000 bp)	456772046	392066999	186491273
Total length	717825918	682211092	415542434
GC (%)	63.9	64.58	65.92
N50^1^	1397	1168	916
L50^2^	112046	145393	128331

The quality of metagenomes were analysed using the program QUAST. The metagenomes were produced by shotgun sequencing of ^13^C-labelled DNA extracted from methanol-enriched wheat rhizosphere soil (wheat), pea plant rhizosphere soil (pea) and unplanted soil (CF) incubated from ^13^C-labelled methanol for 17 days

^1^N50 is the length for which the collection of all contigs of that length or longer covers at least half an assembly

^2^L50 is the number of contigs equal to or longer than N50

Trimmed reads were analysed using Metaphlan 2 (2.0) [43]. Reads were assembled using Megahit (1.1.2) [88] and annotated using myRast (35) [89]. The metagenomes were screened for genes of interest using protein sequences of confirmed function using tblastn in BioEdit [90]. For quantification of the methanol dehydrogenase genes, the assembled and unassembled reads were converted to blast databases and screened using the blastn algorithm and representative sequences for each clade of methanol dehydrogenase-encoding gene (*xoxF1*, *xoxF2*, *xoxF3*, *xoxF4*, *xoxF5*, *mxaF*, *mdh2*). Appropriate stringencies for the BLAST searches were determined by selecting the lowest *e* value that did not yield sequences of the incorrect clade (Additional File 11). Abundance values were normalised to gene length or gene length and read number for the assembled and unassembled metagenome reads respectively. The percentage of bacteria represented in the unassembled read data that possess a methanol dehydrogenase was calculated from the abundance of the methanol dehydrogenase genes divided by the abundance of the housekeeping gene *recA*. The sequences with the highest sequence identity to the identified methanol dehydrogenase sequences from the assembled metagenomes were identified using the blastn algorithm against the NCBI database (Additional File 16).

Contigs were binned into metagenome-assembled genomes (MAGs) using MetaBAT (2.21.1) with the “superspecific” and “veryspecific” algorithms [91]. The completeness, contamination and heterogeneity of these MAGs were assessed using the program CheckM (1.0.13) [92]. The MAGs of sufficient quality binned from the metagenomes (completeness score above 70% and contamination below 10%, meeting currently accepted criteria for medium to high quality MAGs [44]) were also screened for genes of interest using protein sequences of confirmed function using tblastn in BioEdit [90]. Average nucleotide identity (ANI) calculations were performed to assess the similarity of the MAGs Methylophilales ss01 and ss29 to the genomes *Methylovorus* sp. MM2 (NZ_LXUF00000000) and *Methylobacillus* sp. MM3 (NZ_LXTQ00000000) [84].

### Screening of the genomes of members of the *Comamonadaceae* for *xoxF* genes

Three hundred fifteen genomes of members of the *Comamonadaceae* were downloaded from NCBI GenBank. Genomes were screened for the presence of methanol dehydrogenase-encoding genes using local Blast searches (tblastn), using the *xoxF5* sequence of *Variovorax paradoxus* S110 (NZ_ARNA00000000.1) as query in BioEdit [83].

## Supplementary information

**Additional file 1.** 16S rRNA gene profiles of bacteria in unplanted, pea rhizosphere and wheat rhizosphere soils.

**Additional file 2.** Relative abundance (%) of methylotrophic genera in 16S rRNA gene profile of soils.

**Additional file 3.** Phylogeny of mxaF sequences retrieved from pea plant rhizosphere and unplanted soil.

**Additional file 4.** Phylogeny of xoxF5 sequences retrieved from pea plant rhizosphere, wheat plant rhizosphere and unplanted soil.

**Additional file 5.** Phylogeny and relative abundance of xoxF1 sequences retrieved from soil.

**Additional file 6.** Phylogeny and relative abundance of xoxF3 sequences retrieved from soil.

**Additional file 7.** Phylogeny of a xoxF2 sequence retrieved from soil.

**Additional file 8.** Relative abundance of methanol dehydrogenase encoding genes in unplanted and rhizosphere soils.

**Additional file 9.** Diversity indices of the communities in the heavy fraction of methanolenriched rhizosphere and unplanted soils.

**Additional file 10.** Relative abundance of 16s rRNA gene.

**Additional file 11.** Abundance of unique methanol dehydrogenase encoding genes sequences identified in the metagenomes asssembled from methanol-enriched soils.

**Additional file 12.** Diversity of xoxF1 gene sequences retrieved from the heavy fractions of soils enriched with 13C methanol.

**Additional file 13.** Diversity of xoxF4 gene sequences retrieved from the heavy fractions of soils enriched with 13C methanol.

**Additional file 14.** Diversity of xoxF5 gene sequences retrieved from the heavy fractions of soils enriched with 13C methanol.

**Additional file 15.** Diversity of mxaF gene sequences retrieved from the heavy fractions of soils enriched with 13C methanol.

**Additional file 16.** BLAST scoring details of unique mdh2 sequences identified in metagenomes produced from methanol enriched soils.

**Additional file 17.** Details of metagenomes assembled genomes (MAGS) binned from metagenomes assembled from methanol-enriched soils.

**Additional file 18.** Taxonomy of MAGS and the identification of funtional genes involved in C1, nitrogen and sulfur cycling.

**Additional file 19.** Phylogeny of a xoxF3 sequence retrieved from metagenome assembled genome Methylotenera ss03.

**Additional file 20.** Putative and confirmed methylotrophs identified in the pea rhizosphere using multiple approaches.

**Additional file 21.** Putative and confirmed methylotrophs identified in the wheat rhizosphere using multiple approaches.

**Additional file 22.** Putative and confirmed methylotrophs identified in the unplanted soil using multiple approaches.

**Additional file 23.** NMDS plot showing the unweighted unifrac analysis of 16S rRNA gene amplicons produced from DNA extracted from unenriched soils, soils enriched with methanol and rhizosphere soils supplemented with 1000 ppm and 350 ppm carbon dioxide.

**Additional file 24.** Workflow schematic of the DNA-SIP with methanol, the DNA SIP with carbon dioxide and the sequencing and quantification of the methanol dehydrogenase genes and 16S rRNA gene from the soil habitats.

**Additional file 25.** Relative abundance of genera detected as labelled in 16S rRNA gene profiles of the 350 ppmv supplied test groups.

**Additional file 26.** Relative abundance of genera detected as labelled in 16S rRNA gene profiles of the 1000 ppmv supplied test groups.

**Additional file 27.** PCR primers used in this study.

## Data Availability

Amplicon sequence data generated in this study were deposited to sequence read archives (SRA) under project numbers PRJNA533080, PRJNA533036 and PRJNA533035. Metagenome data was deposited to the SRA under project number PRJNA533040. MAGs were uploaded to the SRA under project number PRJNA533039. Clone library sequence data were deposited to NCBI GenBank under accession numbers MN207223-MN207231.
