# Supplementary material for: Impact of plants on the diversity and activity of methylotrophs in soil
Source: Microbiome. 2020 Mar 10;8:31. doi: 10.1186/s40168-020-00801-4 (PMC7065363; doi:10.1186/s40168-020-00801-4)
Supplement: Supplementary file 2 — Additional file 1. 16S rRNA gene profiles of bacteria in unplanted, pea rhizosphere and wheat rhizosphere soils. [file 40168_2020_801_MOESM2_ESM.pdf]

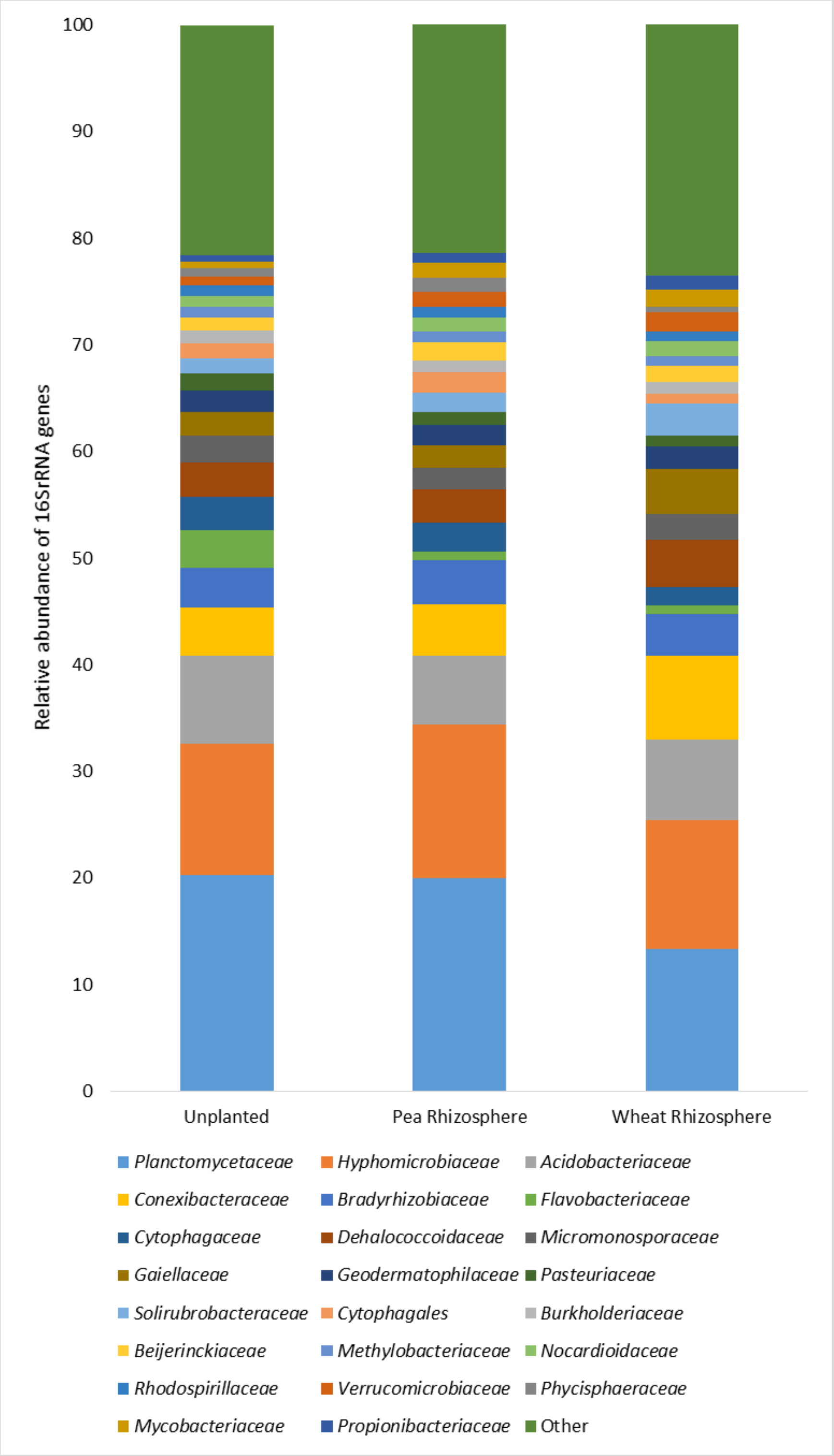

Additional File 1. 16S rRNA gene profiles of bacteria in unplanted, pea rhizosphere and wheat rhizosphere soils.

Sequences were revealed by amplicon sequencing of 16S rRNA gene amplicons retrieved by PCR from DNA extracted from unplanted, pea rhizosphere and wheat rhizosphere soil samples. 16S rRNA gene sequences are presented at the family level. Bacterial families represented at less than 1 % relative abundance are grouped as “Other”.
