# Supplementary material for: Impact of plants on the diversity and activity of methylotrophs in soil
Source: Microbiome. 2020 Mar 10;8:31. doi: 10.1186/s40168-020-00801-4 (PMC7065363; doi:10.1186/s40168-020-00801-4)
Supplement: Supplementary file 3 — Additional file 2. Relative abundance (%) of methylotrophic genera in 16S rRNA gene profile of soils. [file 40168_2020_801_MOESM3_ESM.pdf]

Additional File 2. Relative abundance (%) of methylotrophic genera in 16S rRNA gene profile of soils.

Genus	Putative/ Confirmed	Unplanted soil	Pea Rhizosphere	Wheat Rhizosphere
Hyphomicrobium	Confirmed	4.329	6.219	5.097
Verrucomicrobium	Confirmed	0.715	1.259	1.73
Methylophilus	Confirmed	0.985	1.083	0.615
Beggiatoa	Confirmed	0.187	0.217	0.25
Methylibium	Confirmed	0.232	0.184	0.237
Azosporillium	Confirmed	0.093	0.202	0.182
Methylobacterium	Confirmed	0.168	0.244	0.168
Variovorax	Confirmed	0.102	0.205	0.166
Burkholderia	Confirmed	0.11	0.103	0.136
Methylosinus	Confirmed	0.15	0.106	0.086
Roseomonas	Confirmed	0.04	0.091	0.076
Methylocella	Confirmed	0.076	0.088	0.068
Methylobacillus	Confirmed	0.09	0.088	0.047
Methylocapsa	Confirmed	0.062	0.048	0.044
Sphingomonas	Confirmed	0.025	0.072	0.031
Methylocystis	Confirmed	0.023	0.012	0.026
Methylotenera	Confirmed	0.023	0.021	0.016
Methyloceanibacter	Confirmed	0.008	0.006	0.013
Starkeya	Confirmed	0.017	0.015	0.01
Rhodopseudomonas	Confirmed	0.017	0.018	0.01
Ancylobacter	Confirmed	0.008	0.009	0.01
Xanthobacter	Confirmed	0	0	0.003
Methyloversatilis	Confirmed	0	0	0.003
Methyloligella	Confirmed	0.003	0	0.003
Beijerinckia	Confirmed	0.003	0.003	0.003
Cupriavidus	Confirmed	0	0.003	0
Oharaeibacter	Confirmed	0	0.003	0

Bradyrhizobium	Putative	3.337	3.75	3.586
Flavobacterium	Putative	3.502	0.764	0.796
Gemmatimonas	Putative	0.469	0.42	0.425
Acidovorax	Putative	0.529	0.465	0.237
Leptothrix	Putative	0.074	0.072	0.073
Mesorhizobium	Putative	0.045	0.048	0.055
Pseudomonas	Putative	0.014	0.003	0.013
Verminephrobacter	Putative	0	0.012	0.003
Subaequorebacter	Putative	0.003	0	0.003
Xanthomonas	Putative	0.003	0.003	0
Meganema	Putative	0.003	0.006	0

16S rRNA gene profiles were produced by sequencing of 16S rRNA gene amplicons produced from DNA extracted from pea and wheat plant rhizosphere soils and unplanted soil. Genera were then designated as either confirmed or putative methylotrophs.

Confirmed indicates a genus that contains species confirmed to be capable of methanol oxidation. Putative indicates a genus that contains species that possess methanol dehydrogenase genes.
