# Supplementary material for: Impact of plants on the diversity and activity of methylotrophs in soil
Source: Microbiome. 2020 Mar 10;8:31. doi: 10.1186/s40168-020-00801-4 (PMC7065363; doi:10.1186/s40168-020-00801-4)
Supplement: Supplementary file 4 — Additional file 3. Phylogeny of mxaF sequences retrieved from pea plant rhizosphere and unplanted soil. [file 40168_2020_801_MOESM4_ESM.pdf]

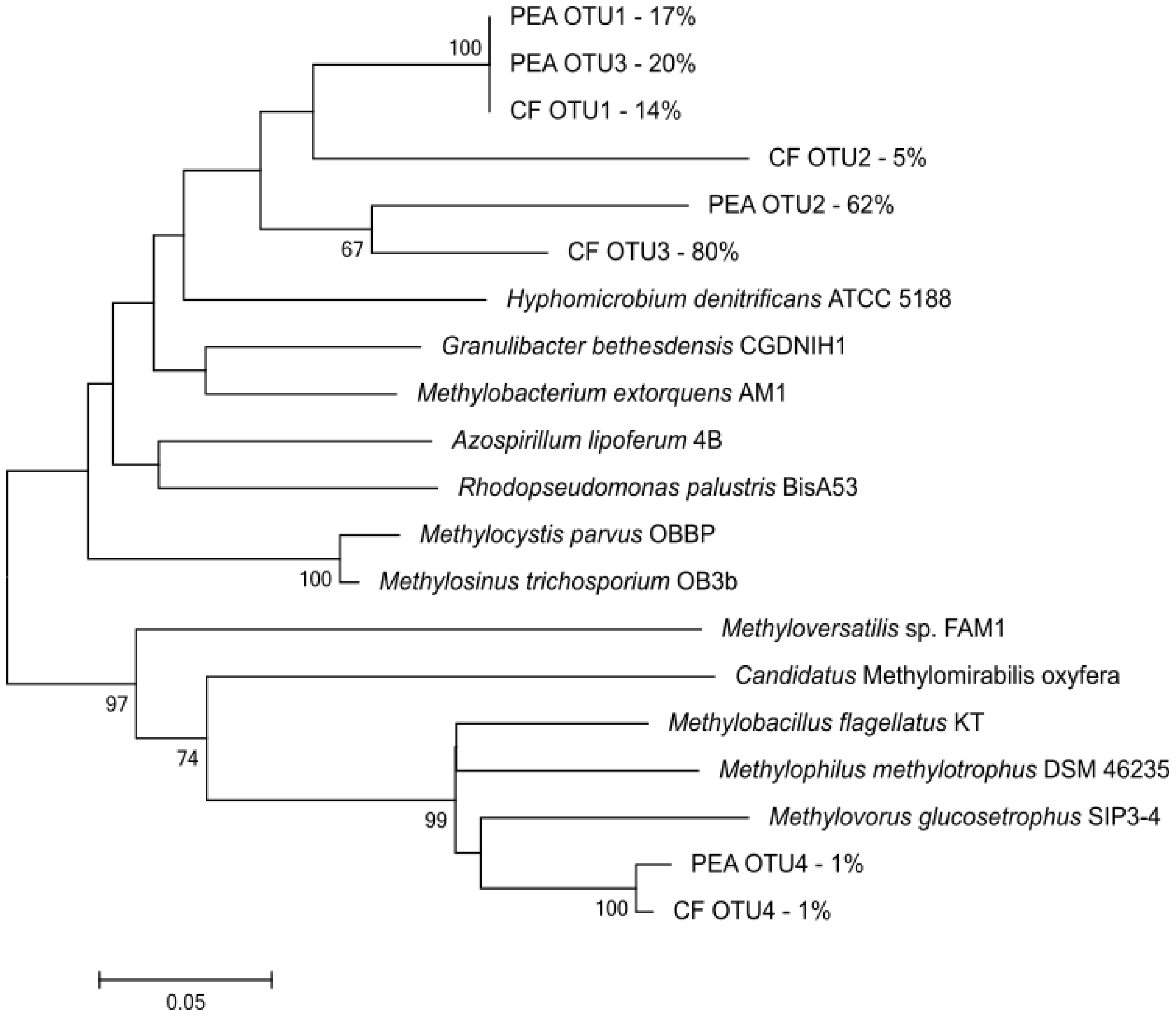

Additional File 3. Phylogeny of *mxaF* sequences retrieved from pea plant rhizosphere and unplanted soil

Phylogenetic tree of *mxaF* sequences retrieved by PCR from DNA extracted from unplanted soil (CF) and pea plant rhizosphere soil (Pea) revealed by 454 amplicon sequencing. Percentages after the OTUs represent relative abundance in the *mxaF* gene profile. Reference gene sequences were selected from the NCBI nucleotide database. Only bootstrap values ≥ 50% (based on 500 replicates) are labelled at branch points. Alignments were produced at the amino acid level with the Muscle algorithm and phylogenetic trees were produced in Mega7 [70] using the neighbour-joining method. There were a total of 135 amino acid residues in the final dataset.
