# Supplementary material for: Impact of plants on the diversity and activity of methylotrophs in soil
Source: Microbiome. 2020 Mar 10;8:31. doi: 10.1186/s40168-020-00801-4 (PMC7065363; doi:10.1186/s40168-020-00801-4)
Supplement: Supplementary file 5 — Additional file 4. Phylogeny of xoxF5 sequences retrieved from pea plant rhizosphere, wheat plant rhizosphere and unplanted soil. [file 40168_2020_801_MOESM5_ESM.pdf]

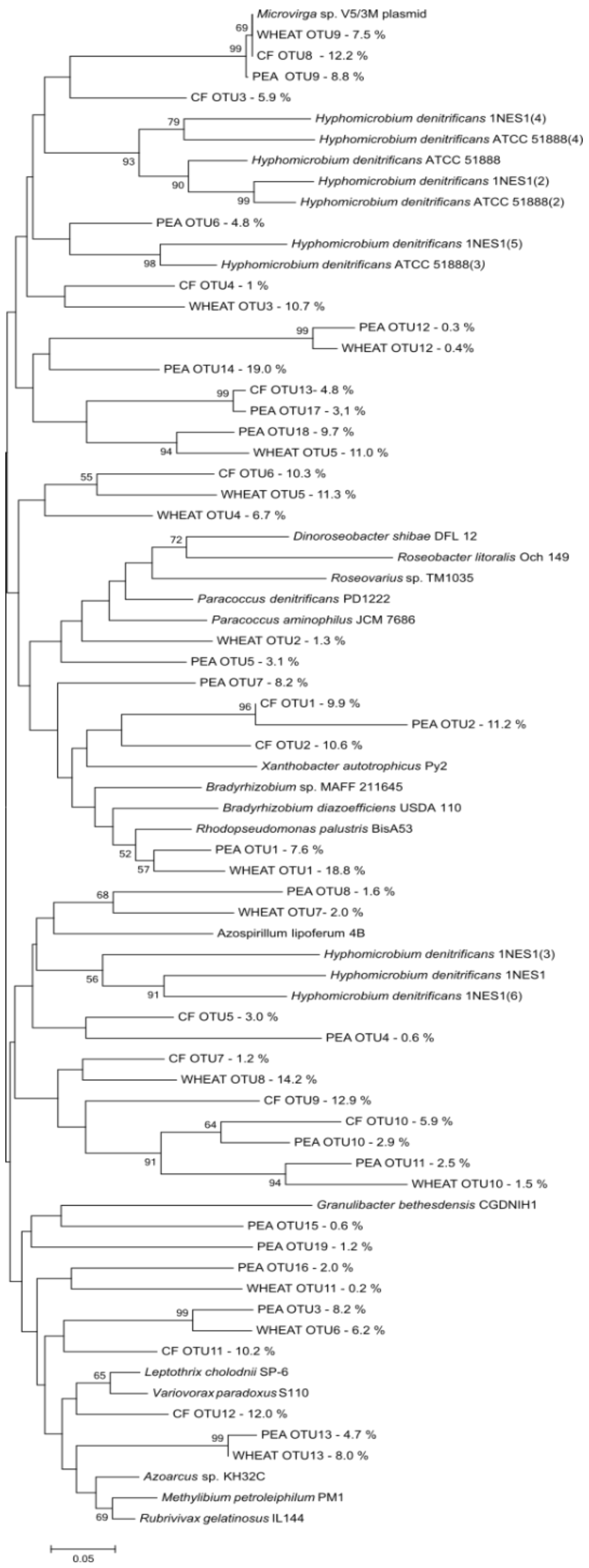
Microvirga sp. V5/3M plasmid
WHEAT OTU9 - 7.5 %
CF OTU8 - 12.2 %
PEA OTU9 - 8.8 %
CF OTU3 - 5.9 %
Hyphomicrobium denitrificans 1NES1(4)
Hyphomicrobium denitrificans ATCC 51888(4)
Hyphomicrobium denitrificans ATCC 51888
Hyphomicrobium denitrificans 1NES1(2)
Hyphomicrobium denitrificans ATCC 51888(2)
PEA OTU6 - 4.8 %
Hyphomicrobium denitrificans 1NES1(5)
Hyphomicrobium denitrificans ATCC 51888(3)
CF OTU4 - 1 %
WHEAT OTU3 - 10.7 %
PEA OTU12 - 0.3 %
WHEAT OTU12 - 0.4%
PEA OTU14 - 19.0 %
CF OTU13- 4.8 %
PEA OTU17 - 3,1 %
PEA OTU18 - 9.7 %
WHEAT OTU5 - 11.0 %
CF OTU6 - 10.3 %
WHEAT OTU5 - 11.3 %
WHEAT OTU4 - 6.7 %
Dinoroseobacter shibae DFL 12
Roseobacter litoralis Och 149
Roseovarius sp. TM1035
Paracoccus denitrificans PD1222
Paracoccus aminophilus JCM 7686
WHEAT OTU2 - 1.3 %
PEA OTU5 - 3.1 %
PEA OTU7 - 8.2 %
CF OTU1 - 9.9 %
PEA OTU2 - 11.2 %
CF OTU2 - 10.6 %
Xanthobacter autotrophicus Py2
Bradyrhizobium sp. MAFF 211645
Bradyrhizobium diazoefficiens USDA 110
Rhodopseudomonas palustris BisA53
PEA OTU1 - 7.6 %
WHEAT OTU1 - 18.8 %
PEA OTU8 - 1.6 %
WHEAT OTU7- 2.0 %
Azospirillum lipoferum 4B
Hyphomicrobium denitrificans 1NES1(3)
Hyphomicrobium denitrificans 1NES1
Hyphomicrobium denitrificans 1NES1(6)
CF OTU5 - 3.0 %
PEA OTU4 - 0.6 %
CF OTU7 - 1.2 %
WHEAT OTU8 - 14.2 %
CF OTU9 - 12.9 %
CF OTU10 - 5.9 %
PEA OTU10 - 2.9 %
PEA OTU11 - 2.5 %
WHEAT OTU10 - 1.5 %
Granulibacter bethesdensis CGDNIH1
PEA OTU15 - 0.6 %
PEA OTU19 - 1.2 %
PEA OTU16 - 2.0 %
WHEAT OTU11 - 0.2 %
PEA OTU3 - 8.2 %
WHEAT OTU6 - 6.2 %
CF OTU11 - 10.2 %
Leptothrix cholodnii SP-6
Variovorax paradoxus S110
CF OTU12 - 12.0 %
PEA OTU13 - 4.7 %
WHEAT OTU13 - 8.0 %
Azoarcus sp. KH32C
Methylibium petroleiphilum PM1
Rubrivivax gelatinosus IL144
0.05

**Additional File 4. Phylogeny of *xoxF5* sequences retrieved from pea plant rhizosphere, wheat plant rhizosphere and unplanted soil**

Phylogenetic tree of *xoxF5* sequences retrieved by PCR from DNA extracted from unplanted soil (CF), pea plant rhizosphere soil (Pea) and wheat plant rhizosphere soil (WHEAT) as revealed by amplicon sequencing. Percentages after the OTUs represent relative abundance in the *xoxF5* gene profile. Reference gene sequences were selected from the NCBI nucleotide database. Only bootstrap values ≥ 50% (based on 500 replicates) are labelled at branch points. Alignments were produced at the amino acid level with the Muscle algorithm and phylogenetic trees were produced in Mega7 [70] using the neighbour-joining method. There were a total of 52 amino acid residues in the final dataset. Multiple *xoxF* gene copies in reference strains are numbered in parentheses.
