# Supplementary material for: Impact of plants on the diversity and activity of methylotrophs in soil
Source: Microbiome. 2020 Mar 10;8:31. doi: 10.1186/s40168-020-00801-4 (PMC7065363; doi:10.1186/s40168-020-00801-4)
Supplement: Supplementary file 6 — Additional file 5. Phylogeny and relative abundance of xoxF1 sequences retrieved from soil. [file 40168_2020_801_MOESM6_ESM.pdf]

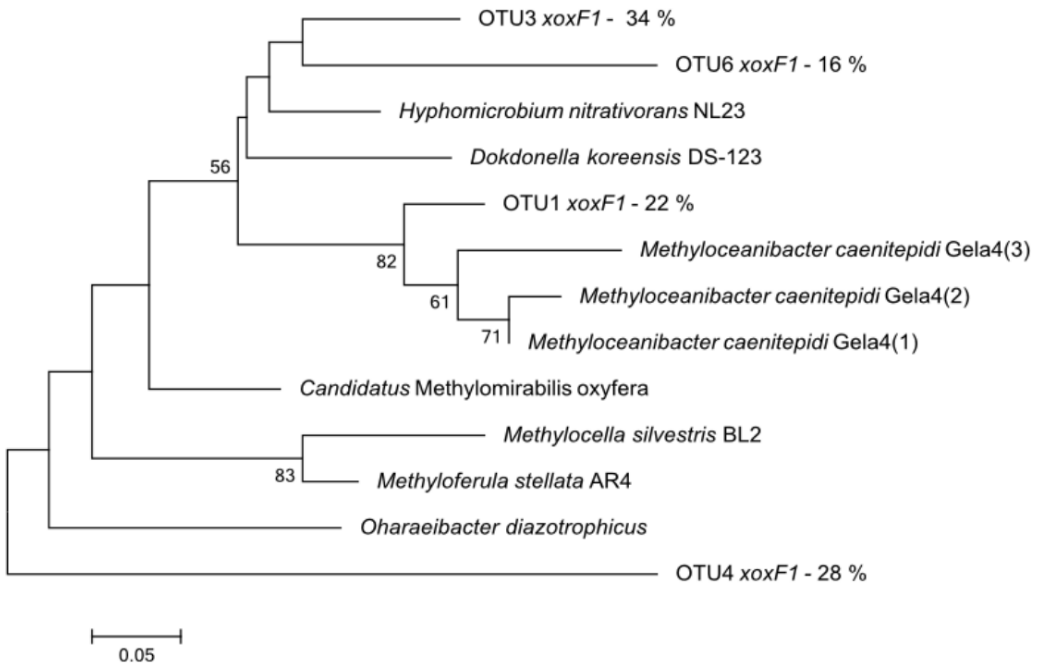

Additional File 5. Phylogeny and relative abundance of *xoxF1* sequences retrieved from soil
Phylogenetic tree of *xoxF1* sequences (OTU1 – OTU4) retrieved by PCR from DNA extracted from unplanted soil as revealed by amplicon sequencing. Reference gene sequences were selected from the NCBI nucleotide database. Only bootstrap values ≥ 50% (based on 500 replicates) are labelled at branch points. Alignments were produced at the amino acid level with the Muscle algorithm and phylogenetic trees were produced in Mega7 [70] using the neighbour-joining method. There were a total of 72 amino acid residues in the final dataset. Multiple *xoxF* gene copies in reference strains are numbered in parentheses.
