# Supplementary material for: Impact of plants on the diversity and activity of methylotrophs in soil
Source: Microbiome. 2020 Mar 10;8:31. doi: 10.1186/s40168-020-00801-4 (PMC7065363; doi:10.1186/s40168-020-00801-4)
Supplement: Supplementary file 7 — Additional file 6. Phylogeny and relative abundance of xoxF3 sequences retrieved from soil. [file 40168_2020_801_MOESM7_ESM.pdf]

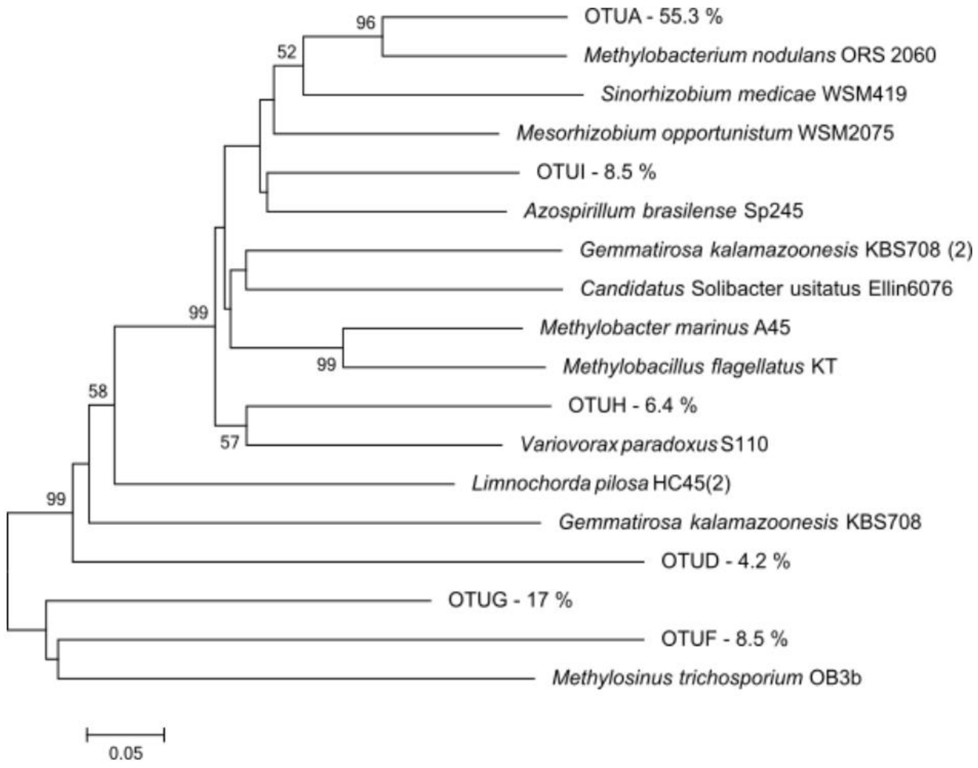

Additional File 6. Phylogeny and relative abundance of *xoxF3* sequences retrieved from soil
Phylogenetic tree of cloned *xoxF3* sequences retrieved by PCR from DNA extracted from unplanted soil DNA as revealed by sequencing. Cloned PCR product are designated as letters. Reference gene sequences were selected from the NCBI nucleotide database. Only bootstrap values ≥ 50% (based on 500 replicates) are labelled at branch points. The alignments was produced at the amino acid level with the Muscle algorithm and phylogenetic trees were produced in Mega7 [70] using the neighbour-joining method. There were a total of 315 amino acid residues in the final dataset.
