# Supplementary material for: Impact of plants on the diversity and activity of methylotrophs in soil
Source: Microbiome. 2020 Mar 10;8:31. doi: 10.1186/s40168-020-00801-4 (PMC7065363; doi:10.1186/s40168-020-00801-4)
Supplement: Supplementary file 8 — Additional file 7. Phylogeny of a xoxF2 sequence retrieved from soil. [file 40168_2020_801_MOESM8_ESM.pdf]

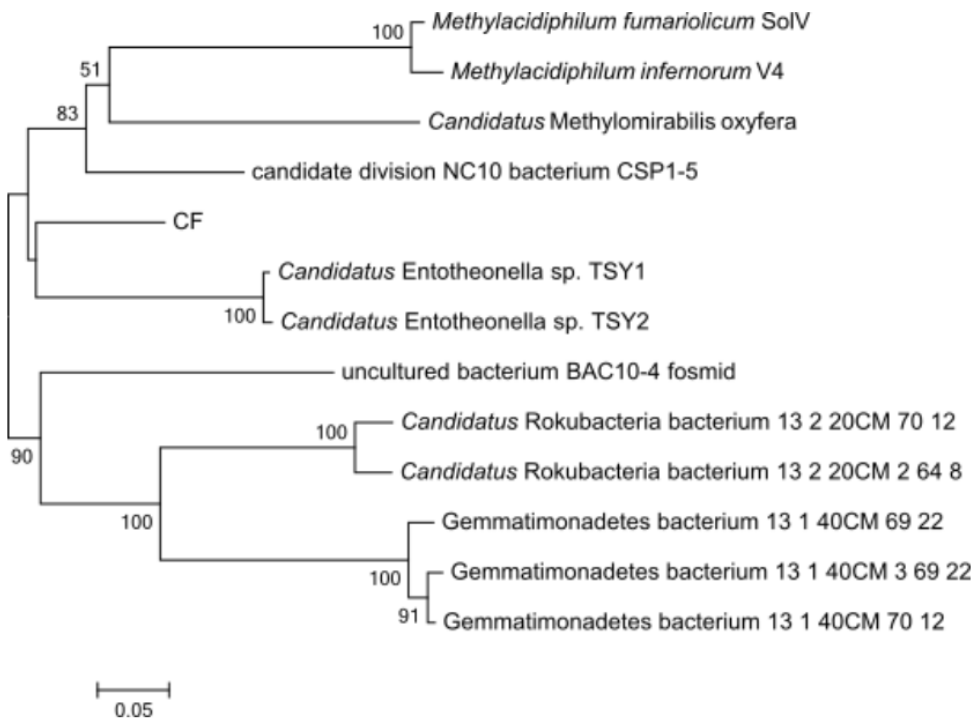

Additional File 7. Phylogeny of a *xoxF2* sequence retrieved from soil.

Phylogenetic tree of a cloned *xoxF2* sequence retrieved by PCR from DNA extracted from unplanted soil as revealed by sequencing. Cloned PCR product is designated “CF”. Reference gene sequences were selected from the NCBI nucleotide database. Only bootstrap values ≥ 50% (based on 500 replicates) are labelled at branch points. Alignments were produced at the amino acid level with the Muscle algorithm and phylogenetic trees were produced in Mega7 [70] with nucleotide sequence data using the neighbour-joining method. Multiple *xoxF* gene copies in reference strains are numbered in parentheses. There were a total of 198 amino acid residues in the final dataset.
