# Supplementary material for: Impact of plants on the diversity and activity of methylotrophs in soil
Source: Microbiome. 2020 Mar 10;8:31. doi: 10.1186/s40168-020-00801-4 (PMC7065363; doi:10.1186/s40168-020-00801-4)
Supplement: Supplementary file 9 — Additional file 8. Relative abundance of methanol dehydrogenase encoding genes in unplanted and rhizosphere soils. [file 40168_2020_801_MOESM9_ESM.pdf]

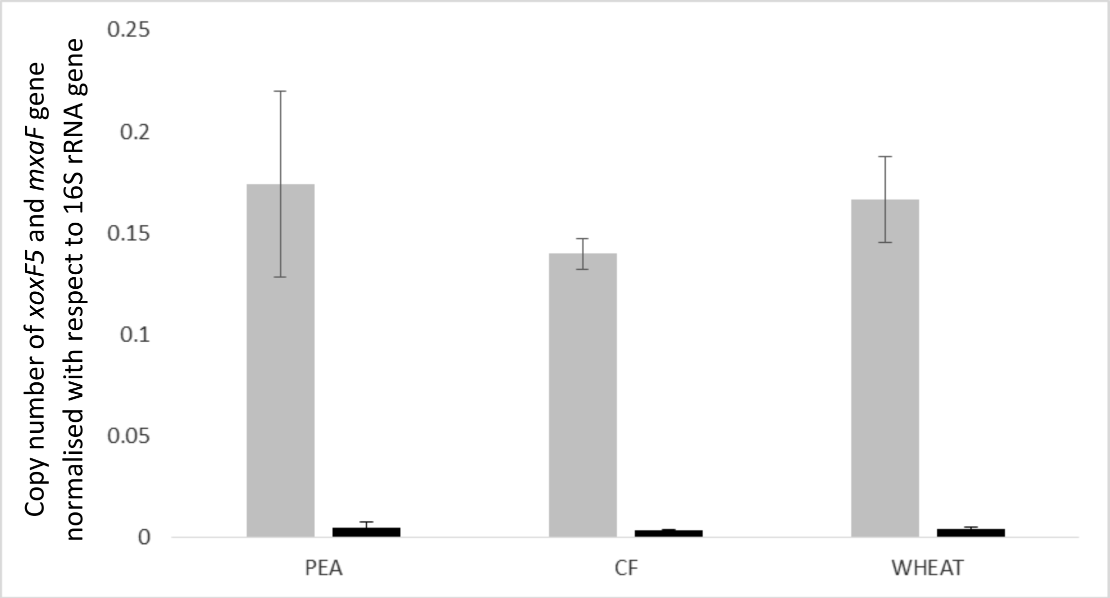

Additional File 8. Relative abundance of methanol dehydrogenase encoding genes in unplanted and rhizosphere soils

The abundance of *mxaF* (black) and *xoxF5* (grey) genes in DNA extracted from unplanted soil (CF), pea rhizosphere soil (PEA) and wheat rhizosphere soil (WHEAT) was estimated by qPCR. *mxaF* and *xoxF* copy numbers with respect to 16S rRNA gene copy number in each sample. Results shown are the average of triplicate samples. Error bars represent standard deviations.
