# Supplementary material for: Impact of plants on the diversity and activity of methylotrophs in soil
Source: Microbiome. 2020 Mar 10;8:31. doi: 10.1186/s40168-020-00801-4 (PMC7065363; doi:10.1186/s40168-020-00801-4)
Supplement: Supplementary file 10 — Additional file 9. Diversity indices of the communities in the heavy fraction of methanolenriched rhizosphere and unplanted soils. [file 40168_2020_801_MOESM10_ESM.pdf]

Additional File 9. Diversity indices of the communities in the heavy fraction of methanol-enriched rhizosphere and unplanted soils

Environment	Timepoint	Sequence type	Community profile	Simpson index 1-D	Shannon index
Unplanted	T1	Amplicon	Labelled	0.04	0.11
Unplanted	T2	Amplicon	Labelled	0.62	1.31
Unplanted	T1	Amplicon	Total	0.93	2.85
Unplanted	T2	Amplicon	Total	0.79	2.20
Unplanted	T2	Metagenome	Total	0.85	2.03
Pea rhizosphere	T1	Amplicon	Labelled	0.75	1.47
Pea rhizosphere	T2	Amplicon	Labelled	0.72	1.68
Pea rhizosphere	T1	Amplicon	Total	0.28	0.88
Pea rhizosphere	T2	Amplicon	Total	0.72	0.54
Pea rhizosphere	T2	Metagenome	Total	0.81	2.19
Wheat rhizosphere	T1	Amplicon	Labelled	0.48	0.93
Wheat rhizosphere	T2	Amplicon	Labelled	0.01	0.03
Wheat rhizosphere	T1	Amplicon	Total	0.68	1.71
Wheat rhizosphere	T2	Amplicon	Total	0.81	2.39
Wheat rhizosphere	T2	Metagenome	Total	0.83	2.36

Shannon diversity index and Simpson diversity index values were calculated from the genera detected in the amplicons and metagenomes produced from the heavy fraction of DNA extracted from the methanol enriched unplanted and pea and wheat rhizosphere soils. Diversity indexes were calculated for either all of the genera detected in an amplicon or the genera identified as labelled in the amplicon datasets.
