# Supplementary figures and images for: Impact of plants on the diversity and activity of methylotrophs in soil
Source: Microbiome. 2020 Mar 10;8:31. doi: 10.1186/s40168-020-00801-4 (PMC7065363; doi:10.1186/s40168-020-00801-4)

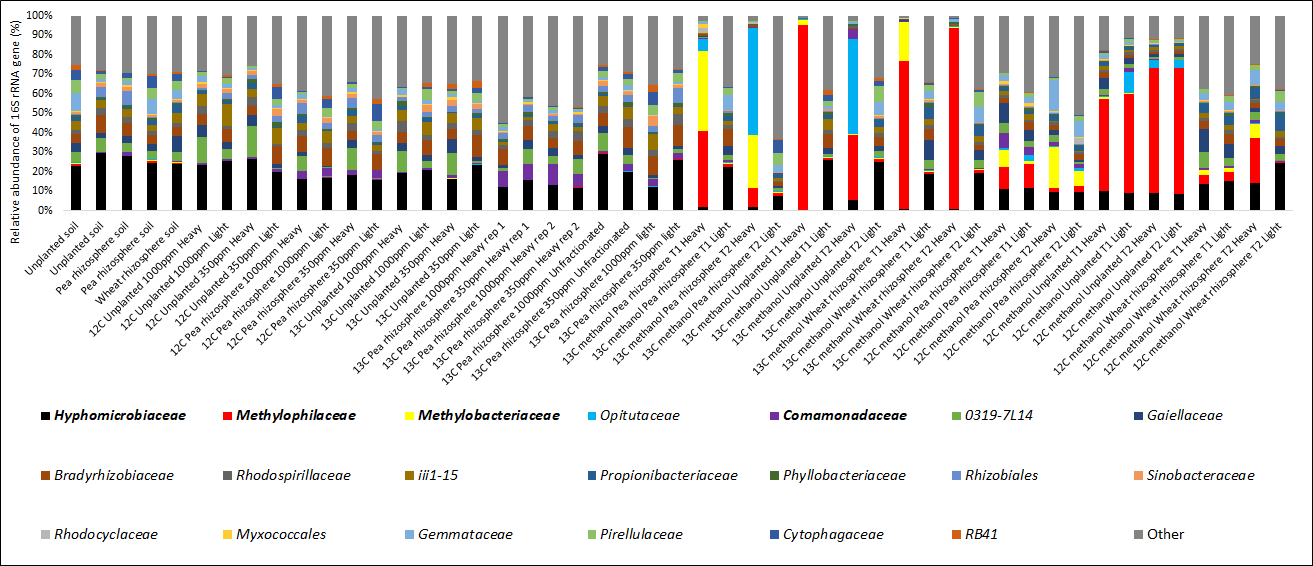

Supplement: Supplementary file 11 — Additional file 10. Relative abundance of 16s rRNA gene. [file 40168_2020_801_MOESM11_ESM.png]

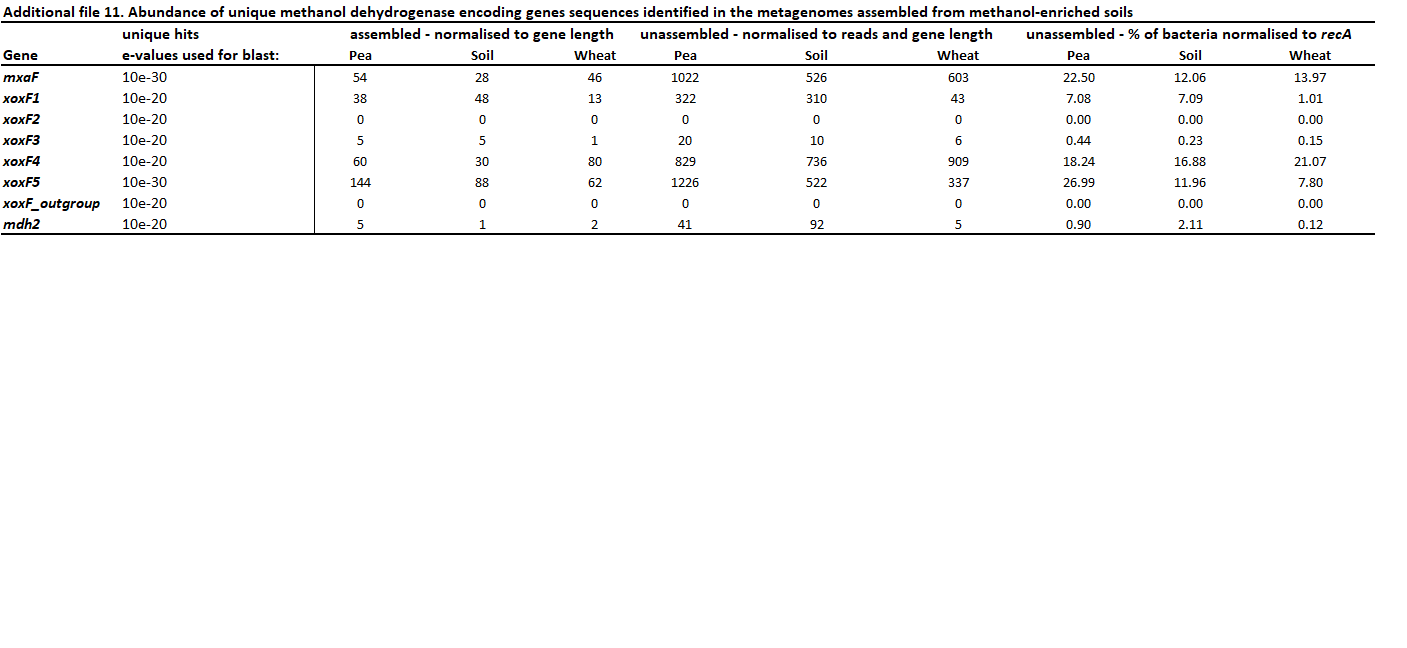

Supplement: Supplementary file 12 — Additional file 11. Abundance of unique methanol dehydrogenase encoding genes sequences identified in the metagenomes asssembled from methanol-enriched soils. [file 40168_2020_801_MOESM12_ESM.png]

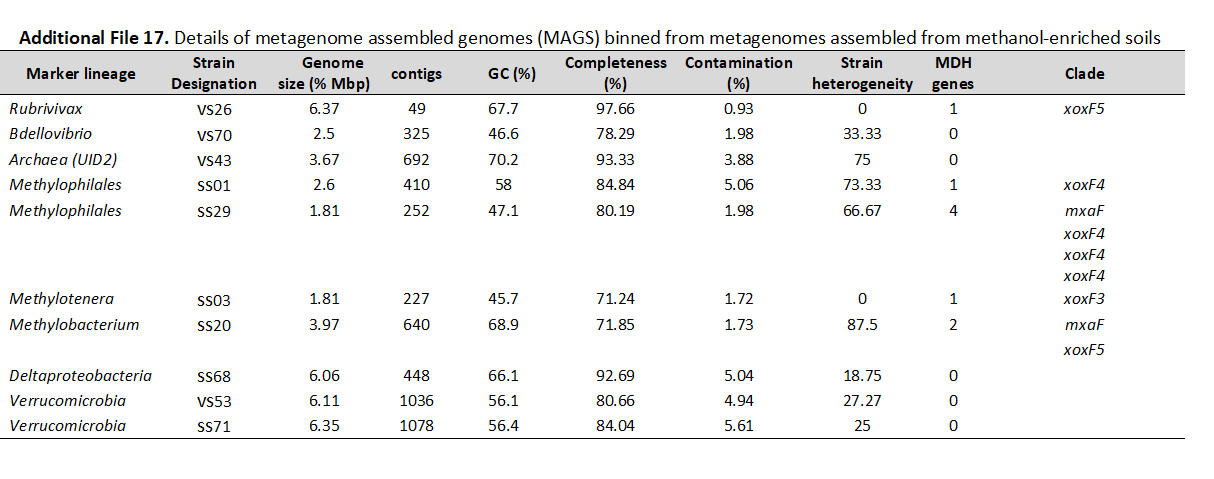

Supplement: Supplementary file 18 — Additional file 17. Details of metagenomes assembled genomes (MAGS) binned from metagenomes assembled from methanol-enriched soils. [file 40168_2020_801_MOESM18_ESM.png]

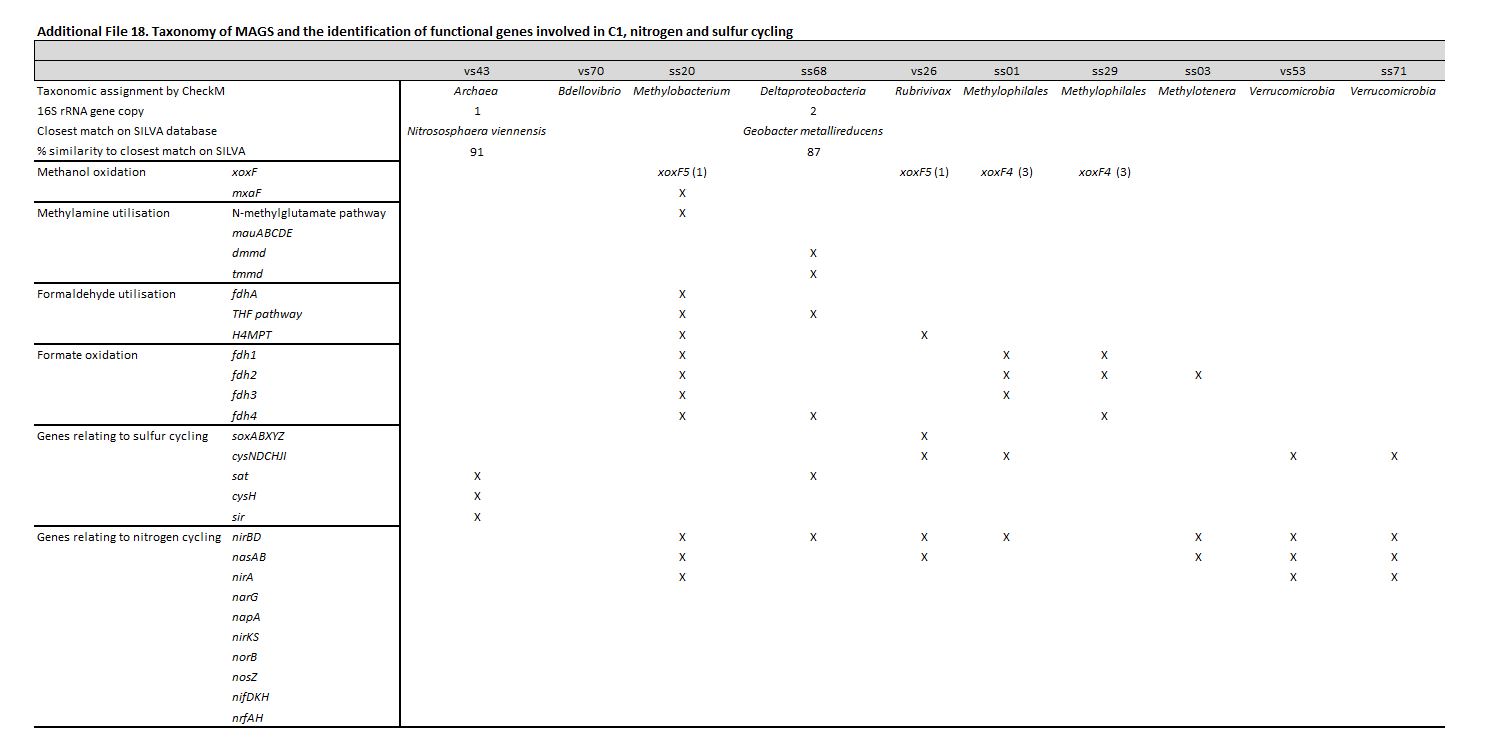

Supplement: Supplementary file 19 — Additional file 18. Taxonomy of MAGS and the identification of funtional genes involved in C1, nitrogen and sulfur cycling. [file 40168_2020_801_MOESM19_ESM.png]

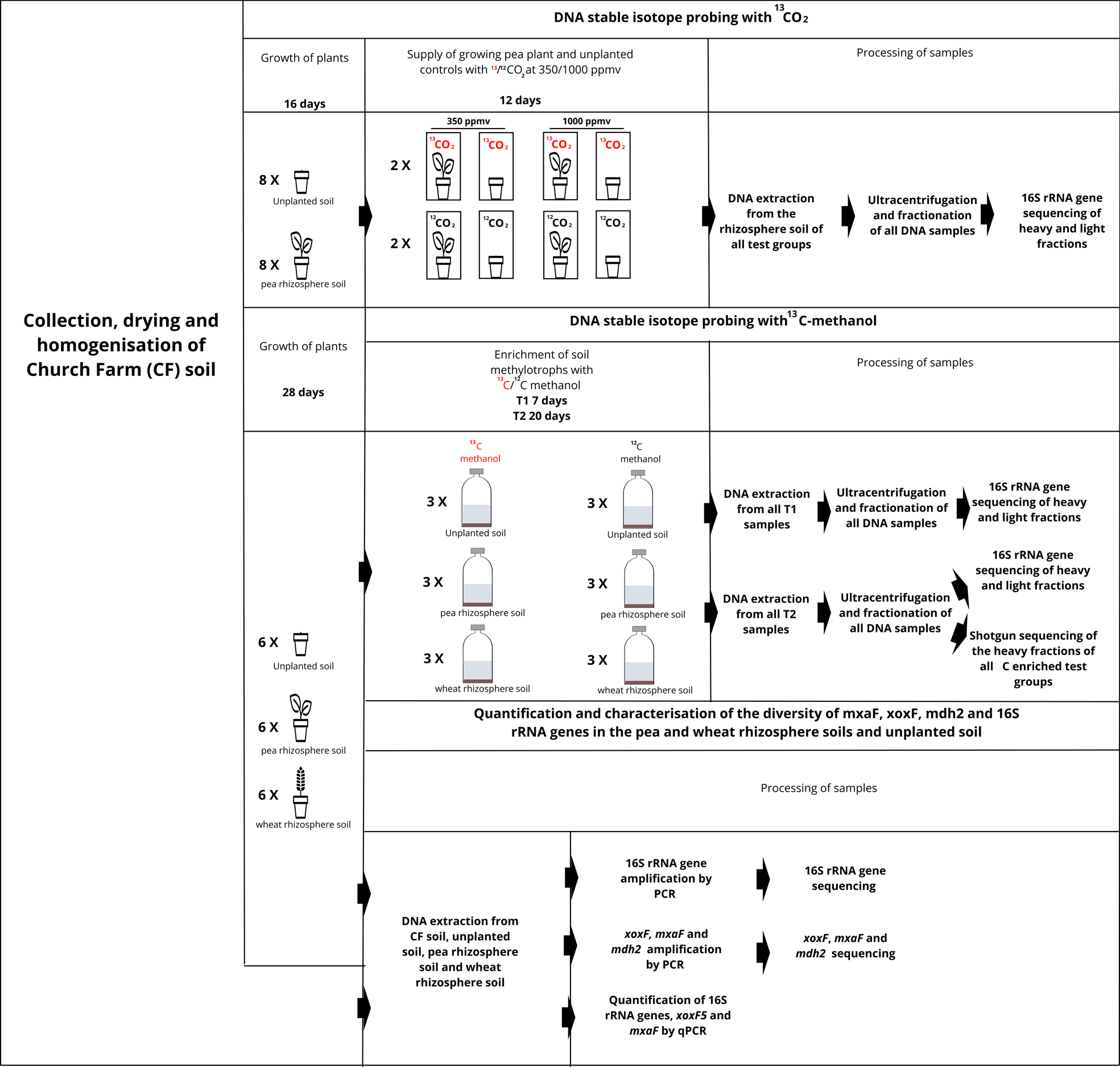

Supplement: Supplementary file 25 — Additional file 24. Workflow schematic of the DNA-SIP with methanol, the DNA SIP with carbon dioxide and the sequencing and quantification of the methanol dehydrogenase genes and 16S rRNA gene from the soil habitats. [file 40168_2020_801_MOESM25_ESM.pdf]
