# Supplementary material for: Impact of plants on the diversity and activity of methylotrophs in soil
Source: Microbiome. 2020 Mar 10;8:31. doi: 10.1186/s40168-020-00801-4 (PMC7065363; doi:10.1186/s40168-020-00801-4)
Supplement: Supplementary file 13 — Additional file 12. Diversity of xoxF1 gene sequences retrieved from the heavy fractions of soils enriched with 13C methanol. [file 40168_2020_801_MOESM13_ESM.pdf]

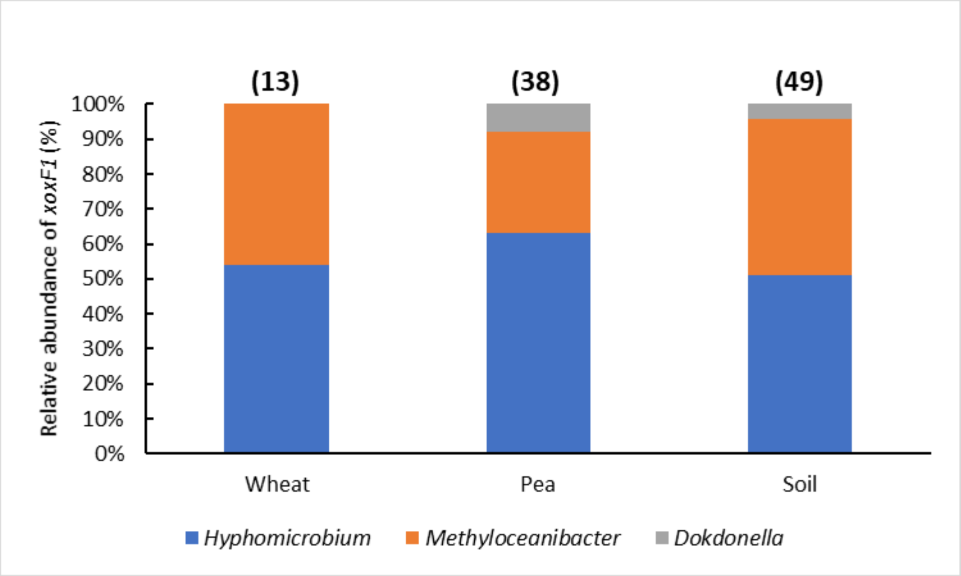

Additional File 12. Diversity of *xoxF1* gene sequences retrieved from the heavy fractions of soils enriched with ^{13}C methanol.

Relative abundance of *xoxF1* sequences affiliated *xoxF1* gene sequences possessed by characterised and genome sequenced organisms. These sequences were retrieved from the assembled reads of metagenomes produced from the heavy fractions of DNA extracted from Unplanted (Soil), pea rhizosphere (Pea) and wheat rhizosphere (Wheat) soil samples enriched with ^{13}C methanol for 17 days (T2) in a DNA-SIP experiment. The number of contigs containing *xoxF1* sequences is displayed in brackets above the columns.
