# Supplementary material for: Impact of plants on the diversity and activity of methylotrophs in soil
Source: Microbiome. 2020 Mar 10;8:31. doi: 10.1186/s40168-020-00801-4 (PMC7065363; doi:10.1186/s40168-020-00801-4)
Supplement: Supplementary file 16 — Additional file 15. Diversity of mxaF gene sequences retrieved from the heavy fractions of soils enriched with 13C methanol. [file 40168_2020_801_MOESM16_ESM.pdf]

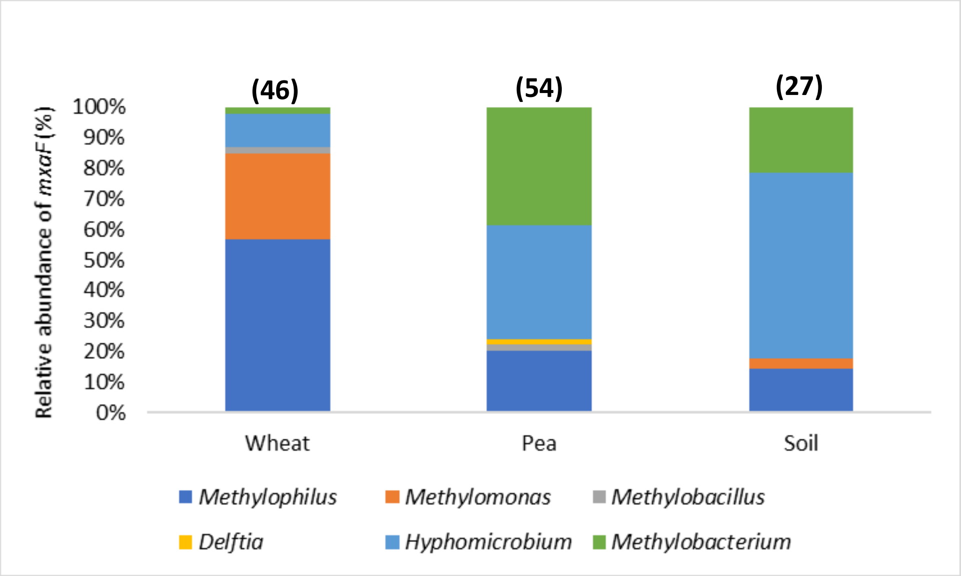

Additional File 15. Diversity of *mxaF* gene sequences retrieved from the heavy fractions of soils enriched with ^{13}C methanol.

Relative abundance of taxa based on *mxaF* gene sequences retrieved from the assembled reads of the metagenomes produced from the heavy fractions of DNA extracted from Unplanted (Soil), pea rhizosphere (Pea) and wheat rhizosphere (Wheat) soil samples enriched with ^{13}C methanol for 17 days (T2) in a DNA-SIP experiment. The number of contigs containing *mxaF* sequences is displayed in brackets above the columns.
