# Supplementary material for: Impact of plants on the diversity and activity of methylotrophs in soil
Source: Microbiome. 2020 Mar 10;8:31. doi: 10.1186/s40168-020-00801-4 (PMC7065363; doi:10.1186/s40168-020-00801-4)
Supplement: Supplementary file 20 — Additional file 19. Phylogeny of a xoxF3 sequence retrieved from metagenome assembled genome Methylotenera ss03. [file 40168_2020_801_MOESM20_ESM.pdf]

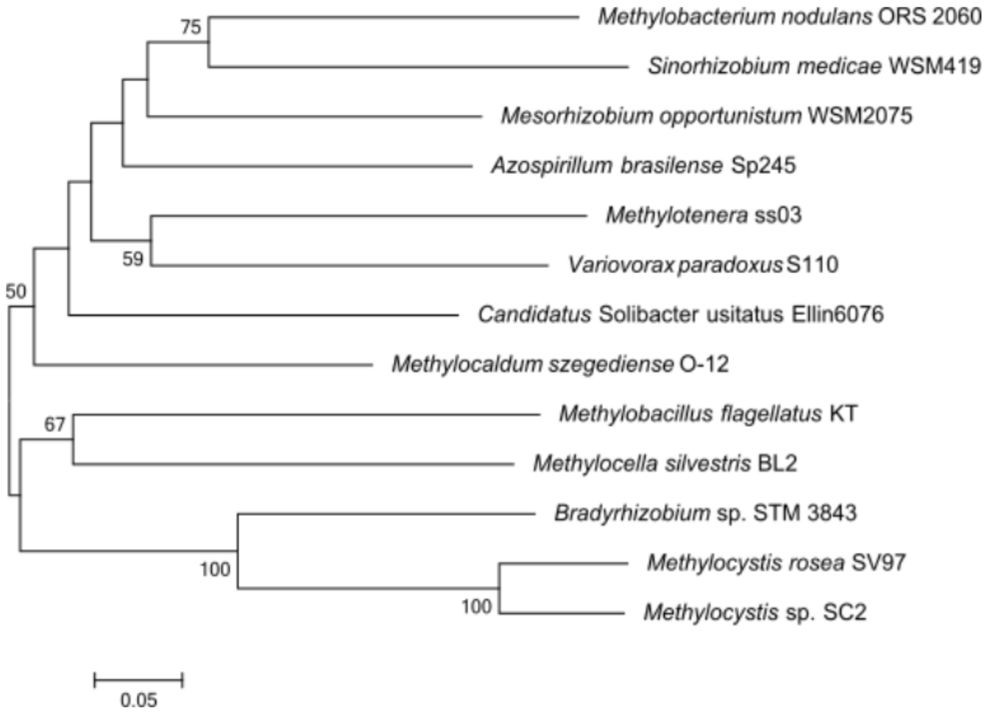

Additional File 19. Phylogeny of a *xoxF3* sequence retrieved from metagenome assembled genome *Methylotenera* ss03.

Phylogenetic tree of *xoxF3* sequences retrieved from the MAG *Methylotenera* ss03. Reference gene sequences were selected from the NCBI nucleotide database. Only bootstrap values ≥ 50% (based on 500 replicates) are labelled at branch points. Alignments were produced at the amino acid level with the Muscle algorithm and phylogenetic trees were produced in Mega7 [70] with nucleotide sequence data using the neighbour-joining method. There was a total of 193 amino acid residues in the final dataset.
