# Supplementary material for: Impact of plants on the diversity and activity of methylotrophs in soil
Source: Microbiome. 2020 Mar 10;8:31. doi: 10.1186/s40168-020-00801-4 (PMC7065363; doi:10.1186/s40168-020-00801-4)
Supplement: Supplementary file 24 — Additional file 23. NMDS plot showing the unweighted unifrac analysis of 16S rRNA gene amplicons produced from DNA extracted from unenriched soils, soils enriched with methanol and rhizosphere soils supplemented with 1000 ppm and 350 ppm carbon dioxide. [file 40168_2020_801_MOESM24_ESM.pdf]

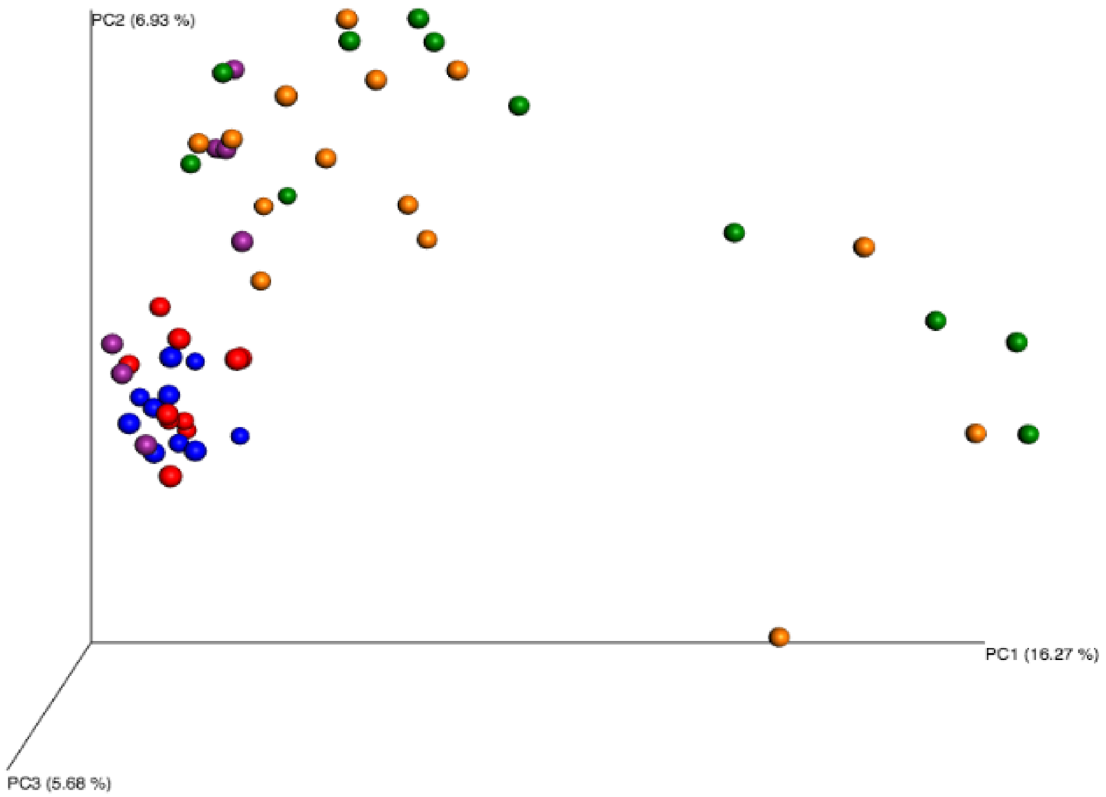

(10) CO_2 - 1000 ppm
(14) Methanol Enrichment - T1
(7) Unenriched soils
(10) CO_2 - 350 ppm
(12) Methanol Enrichment - T2

Additional file 23. NMDS plot showing the unweighted unifrac analysis of 16S rRNA gene amplicons produced from DNA extracted from unenriched soils, soils enriched with methanol and rhizosphere soils supplemented with 1000 ppm and 350 ppm carbon dioxide
