# Supplementary material for: Impact of plants on the diversity and activity of methylotrophs in soil
Source: Microbiome. 2020 Mar 10;8:31. doi: 10.1186/s40168-020-00801-4 (PMC7065363; doi:10.1186/s40168-020-00801-4)
Supplement: Supplementary file 26 — Additional file 25. Relative abundance of genera detected as labelled in 16S rRNA gene profiles of the 350 ppmv supplied test groups. [file 40168_2020_801_MOESM26_ESM.pdf]

Additional File 25. Relative abundance of genera detected as labelled in 16S rRNA gene profiles of the 350 ppmv supplied test groups

	Pea rhizosphere				Unplanted soil			
Genera	^{13}C heavy fraction	^{13}C light fraction	^{12}C heavy fraction	^{12}C light fraction	^{13}C heavy fraction	^{13}C light fraction	^{12}C heavy fraction	^{12}C light fraction
Roseomonas	0.25	0.06	0.08	0.15	0.64	0	0	0.29
Streptosporangium	0.11	0	0	0	0.37	0	0	0
Aquabacterium	0.19	0.03	0.04	0	0.18	0.08	0	0
Chitinophaga	0.06	0	0	0	0.09	0	0	0.15
Solitalea	0.06	0	0	0.11	0.18	0	0	0
Stella	0.23	0.10	0	0	0.46	0	0.07	0.15
Sorangium	0.23	0.06	0.08	0	0.46	0	0.07	0
Herbaspirillum	0.05	0	0	0	0.28	0	0	0
