# Supplementary material for: Impact of plants on the diversity and activity of methylotrophs in soil
Source: Microbiome. 2020 Mar 10;8:31. doi: 10.1186/s40168-020-00801-4 (PMC7065363; doi:10.1186/s40168-020-00801-4)
Supplement: Supplementary file 27 — Additional file 26. Relative abundance of genera detected as labelled in 16S rRNA gene profiles of the 1000 ppmv supplied test groups. [file 40168_2020_801_MOESM27_ESM.pdf]

Additional File 26. Relative abundance of genera detected as labelled in 16S rRNA gene profiles of the 1000 ppmv supplied test groups

	Pea rhizosphere				Unplanted soil			
Genera	^{13}C heavy fraction	^{13}C light fraction	^{12}C heavy fraction	^{12}C light fraction	^{13}C heavy fraction	^{13}C light fraction	^{12}C heavy fraction	^{12}C light fraction
Phenylobacterium	0.30	0	0.13	0.07	0.06	0	0	0
Micromonospora	0.11	0	0	0	0.13	0	0	0
Ilumatobacter	0.55	0	0.27	0.11	0.64	0	0.15	0
