# Supplementary material for: Impact of plants on the diversity and activity of methylotrophs in soil
Source: Microbiome. 2020 Mar 10;8:31. doi: 10.1186/s40168-020-00801-4 (PMC7065363; doi:10.1186/s40168-020-00801-4)
Supplement: Supplementary file 28 — Additional file 27. PCR primers used in this study. [file 40168_2020_801_MOESM28_ESM.pdf]

Additional File 27. PCR primers used in this study

Primers	Target gene	Sequence	Reference	Positive Control	Amplicon length (bp)	Annealing temp (°C)	Cycles
27F MOD	16S rRNA gene	AGRGTTTGATCMTGGCTCAG			492	60	30
519R MODBIO		GTNTTACNGCGGCKGCTG					
xoxF1F	*xoxF1*	TAYGCCGAYGGCAAGSTGST	(Taubert *et al.*, 2015)	*Methylocella silvestris* BL2	600	65- 55 Touchdown	30
xoxF1R		CCGTCRTARTCCCAYTGRTCGAA					
xoxF2F	*xoxF2*	GGCYTAYCAGATGACBCCNTGG		Confirmed *xoxF2* containing clone	620	62- 52 Touchdown	30
xoxF2R		GCCTTRAACCAKCCRTCCA					
xoxF3F	*xoxF3*	GGHGAGWCCATSACVATGGC		*Methylocella silvestris* BL2	1000	62- 52 Touchdown	30
xoxF3R		TCCATSGTKCCGTAGAA					
xoxF4F	*xoxF4*	TTYCCHAAYAACGTNTAYGC		*Methylobacillus flagellatus* KT	660	58- 48 Touchdown	30
xoxF4R		GGRTTRCCHGTHCCGTAGTA					
xoxF5F	*xoxF5*	GAYGAVTGGGAYTWYGACGG		*Methylocella silvestris* BL2	370-390	62- 52 Touchdown	30
xoxF5R		GGYTCVTARTCCATRCA					
1003F	*mxaF*	GCGGCACCAACTGGGGCTGGT	(Neufeld, *et al.*,2007)	*Methylobacterium extorquens* AM1	552	65- 55 Touchdown	30
1555R		CATGAABGGCTCCCARTCCAT					
M13F	Insert-flanking regions of pGEM®-T Easy Vector	GTAAAACGACGGCCAG	Invitrogen		Insert size + 200bp	56	35
M13R		CAGGAAACAGCTATGAC					
mdh2F	*mdh2*	TGGCAGACCGCSTCGTTCGA	This work	*Methyloversatilis discipiluorum* LF1	516	52	35
mdh2R		CAGTTGGTGCCGCCSAGGAA					
